# An Electrolyte with Elevated Average Valence for Suppressing the Capacity Decay of Vanadium Redox Flow Batteries

**DOI:** 10.1021/acscentsci.2c01112

**Published:** 2022-12-23

**Authors:** Zhenyu Wang, Zixiao Guo, Jiayou Ren, Yiju Li, Bin Liu, Xinzhuang Fan, Tianshou Zhao

**Affiliations:** † Department of Mechanical and Aerospace Engineering, The Hong Kong University of Science and Technology, Clear Water Bay, Kowloon, Hong Kong SAR999077, People’s Republic of China; ‡ Department of Mechanical and Energy Engineering, Southern University of Science and Technology, Shenzhen518055, People’s Republic of China

## Abstract

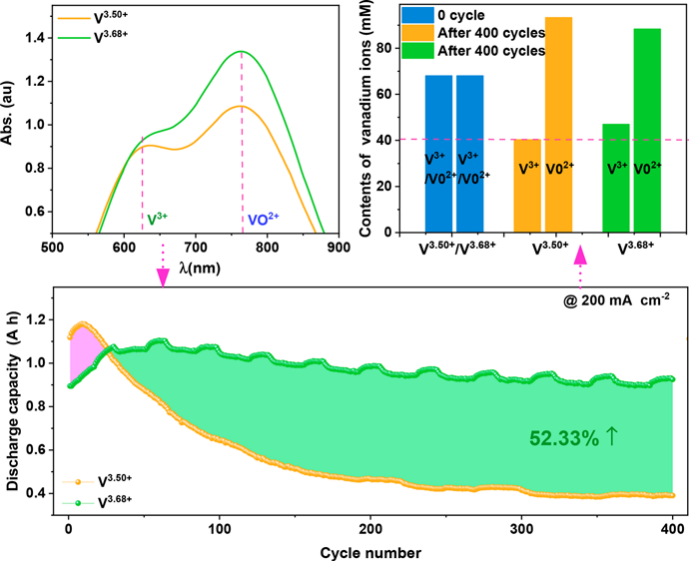

Nafion series membranes are widely used in vanadium redox flow batteries (VRFBs). However, the poor ion selectivity of the membranes to vanadium ions, especially for V^2+^, results in a rapid capacity decay during cycling. Although tremendous efforts have been made to improve the membrane’s ion selectivity, increasing the ion selectivity without sacrificing the proton conductivity is still a challenging issue. In this work, instead of focusing on enhancing the membranes’ ion selectivity, we develop an efficient valence regulation strategy to suppress the capacity decay caused by the crossover of V^2+^ in VRFBs. Despite the discharge capacity of the VRFB with the elevated average valence electrolytes (V^3.68+^) being slightly lower than that with commercial electrolytes (V^3.50+^) in the first 35 cycles, the accumulated discharge capacity in 400 cycles is improved by 52.33%. Moreover, this method is efficient, is easy to scale up, and provides deep insights into the capacity decay mechanism of VRFBs.

## Abstract 2

Compared with the commercial electrolytes (V^3.50+^), the accumulated discharge capacity in 400 cycles of a VRFB with the elevated average valence electrolytes (V^3.68+^) is improved by 52.33%.

## Introduction

A vanadium redox flow battery (VRFB) is one of the most promising large-scale energy storage technologies due to its high safety, long lifespan, easy scalability, and flexible design, which makes it viable for large-scale energy storage systems (especially for those larger than 1 MW) in the next 10–15 years.^1,2^ However, rapid capacity decay is still an intractable issue in long-term cycling for VRFBs. In VRFBs, the redox couples of VO^2+^/VO_2_^+^ and V^2+^/V^3+^ are adopted as the active species on the positive and negative sides, separated by an ion exchange membrane. Ideally, the V^3+^ (V^2+^) content in the anolyte should be equal to that of VO^2+^ (VO_2_^+^) in the catholyte to ensure that the electrolytes have the highest available capacity. However, the electrolyte state gradually deteriorates with cycling (such as volume imbalance, valence imbalance, and concentration imbalance) due to the membrane’s unideal ion selectivity, resulting in rapid capacity decay.

Nafion series membranes are the most widely used proton exchange membranes in VRFBs. However, V^2+^ has a much higher diffusion rate across the Nafion series membranes than other vanadium ions (V^2+^ > VO^2+^ > VO_2_^+^ > V^3+^),^3^ which results in the net flux of V^2+^ transport from the negative to the positive side.^4,5^ Meanwhile, side reactions occur after V^2+^ transports to the positive side:^6^
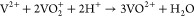
1
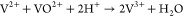
2
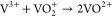
3

Overall, no matter whether V^2+^ reacts with VO_2_^+^ directly (eq 1) or reacts with VO^2+^ first and then reacts with VO_2_^+^ (eqs 2 and 3), 1 mol of V^2+^ consumes 2 mol of VO_2_^+^ after final crossovers. As a result, the concentration and volume (1 mol of V^2+^ crossover combined with 6 mol of H_2_O) of the electrolyte on the positive side are finally increased, but the VO_2_^+^ is scanty on the positive side and continues to intensify with cycling.^7^ Oppositely, the negative electrolyte volume decreases during the cycling, but some V^2+^ cannot be used due to the scanty VO_2_^+^, leading to the unavailable V^2+^ accumulating on the negative side. Such a volume imbalance, valence imbalance, and concentration imbalance of the electrolytes on the positive and negative sides significantly reduce the capacity of VRFBs.

Numerous works have focused on improving the ion selectivity of Nafion series membranes in VRFBs to suppress the capacity decay. Some researchers composited Teflon,^8^ polybenzimidazole (PBI),^9^ and polypropylene (PP)^10^ with Nafion or modified the Nafion membranes with microporous materials such as zeolites^11^ and oriented graphene oxide (GO)^12^ to enhance the membranes’ ion selectivity. However, the delamination of such composite structures^13^ is usually inevitable, and the tradeoff between ion selectivity and proton conductivity remains challenging.^14^

Moreover, introducing excess electrolytes on the negative side^15^ or redividing the electrolytes after remixing the positive and negative electrolytes^16^ has been applied to partially restore the capacity of VRFBs. However, these methods can only partially balance the contents and valence states of electrolytes on the positive and negative sides but are ineffective in reducing the crossover and capacity decay rate.

Even though the electrolyte utilization of VRFBs can be enhanced to some extent by optimizing the sulfuric acid concentration,^18^ adopting a mixed acid system,^19,20^ and increasing the operating temperature (with some organic/inorganic additives),^21−24^ such research is mainly focused on improving the energy efficiency of VRFBs. There has been no research on improving the capacity retention of VRFBs by optimizing the configuration of electrolytes.

In this work, by exploring the capacity decay mechanisms of VRFBs with Nafion membranes in depth, we developed an electrolyte with elevated average valence to eliminate the unavailable V^2+^ accumulation on the negative side during cycling by generating surplus VO_2_^+^ at the first charge/discharge cycle. Although elevating the average valence of electrolytes sacrifices the capacity of VRFBs in the first 35 cycles to a certain extent, the elimination of unavailable V^2+^ during cycling can greatly decrease the crossover of V^2+^ and significantly improve the cycling performance. Notably, the accumulated discharge capacity of VRFBs with V^3.68+^ in 400 cycles is improved by 52.33% from 256.97 to 391.43 Ah in comparison to that with commercial balance valence electrolyte (V^3.50+^).

## Results and Discussion

### Vanadium Ion Concentration Varies with Cycling

**Figure 1:**
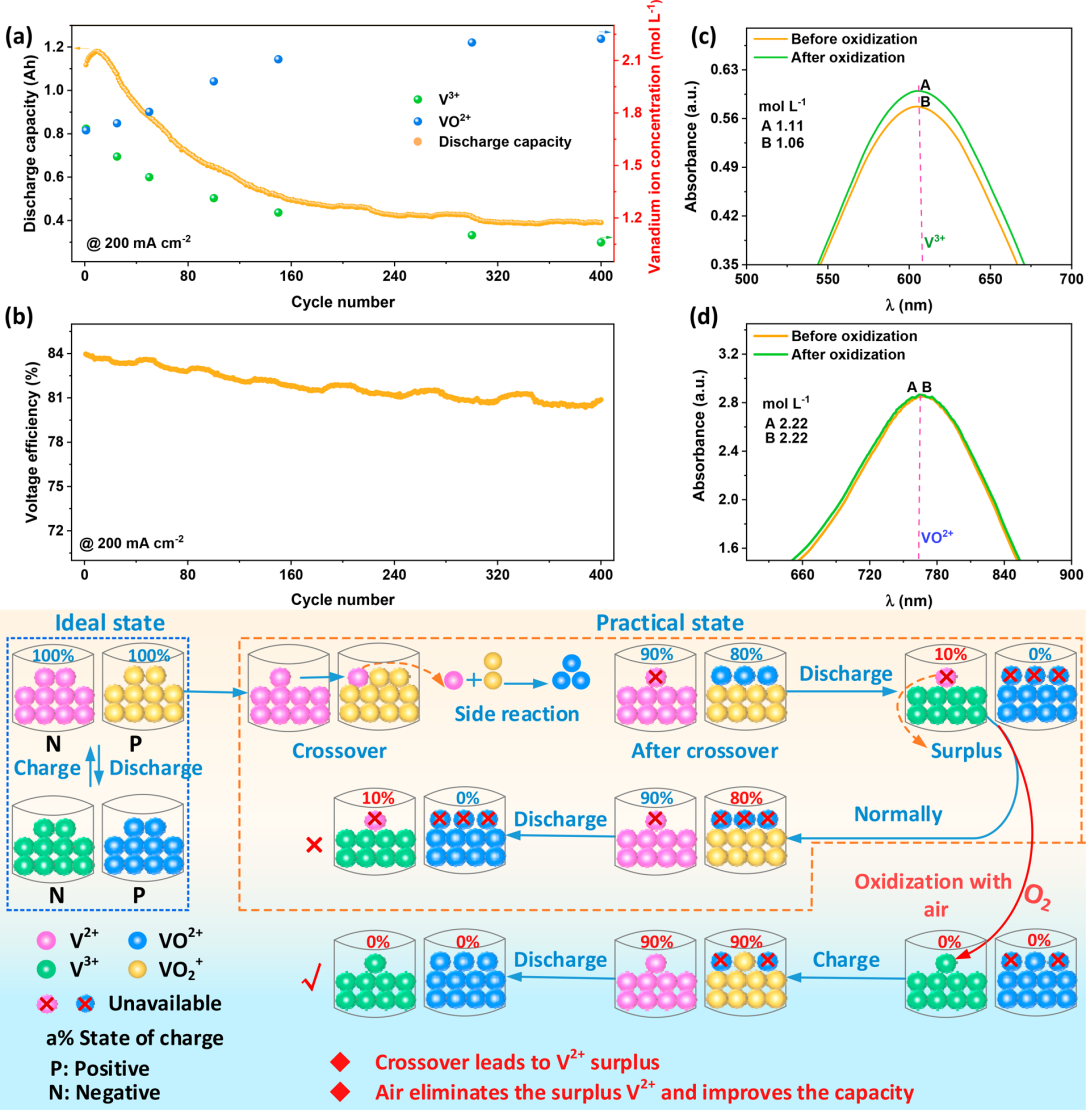
(a) Change in capacity and concentration of V^3+^ and VO^2+^ with cycle number in VRFB. (b) Change in voltage efficiency with cycle number. (c) V^3+^ and (d) VO^2+^ concentrations before and after oxidation with air. (e) Schematic diagram of the evolution of vanadium ions during the charge/discharge processes.

As was mentioned above, the concentration of different vanadium ions varies with cycling due to their different diffusion rates across the Nafion membranes. Herein, the discharge capacity of VRFBs with commercial electrolytes (V^3.50+^) and the concentration of different vanadium ions varying with cycling was tested (detailed information is shown in the Supporting Information), as shown in Figure 1. In this work, we only measured the concentration of VO^2+^ and V^3+^, which can reflect the total contents of vanadium ions after fully discharging and oxidizing V^2+^ with air (Figure S4).

Figure 1a shows that the concentration of VO^2+^ increases rapidly with cycling in the first 150 cycles and then grows slowly, while the concentration of V^3+^ decreases in an opposite trend, which agrees with previous reports.^12,13^ Moreover, the variation of vanadium ion concentration results in viscosity differences between the positive and negative sides (Figure S5). The viscosity differences increase with cycling and finally arrive at a dynamic balance between diffusion and convection, where the discharge capacity of VRFBs almost keeps a stable state^25^ (at about 200 cycles). Besides, the voltage efficiency (VE) of VRFBs decreases slightly with the cycle number (Figure 1b), while the VE and discharge capacity are fully recovered after refreshing the electrolyte (Figure S6). This indicates that the decay of VE and discharge capacity can be ascribed to the increasing concentration loss caused by electrolyte imbalance during cycling.^26^ In other words, it can be deduced that the capacity decay of VRFBs is exclusively caused by the crossover of vanadium ions.

As was mentioned above, VO^2+^ is the only vanadium ion on the positive side after being fully discharged, while both V^2+^ and V^3+^ exist in the negative electrolytes in the VRFB after crossover. However, it is difficult to detect the remainder of V^2+^ after fully discharging because the absorbance signal of V^2+^ (562 nm) is weak and close to the peak of V^3+^ (610 nm) in the UV spectrum.^25^ Therefore, the generation of unavailable V^2+^ with cycling is confirmed by testing the concentration variation of V^3+^ before and after oxidation in the air. As shown in Figure 1c, the content of V^3+^ increases profoundly after oxidizing the electrolytes with air, while the concentration of VO^2+^ almost remains the same (Figure 1d), signifying the existence of the unavailable V^2+^ on the negative side during cycling. Notably, the concentration difference of V^3+^ displayed in Figure 1c is smaller than its actual value of accumulated V^2+^ due to the unavailable oxidation of V^2+^ with air during the UV test.

Thus, for the VRFBs with a commercial electrolyte (V^3.50+^), the volume and content of the positive electrolytes increase but VO_2_^+^ is scanty due to the crossover of V^2+^ after long-term cycling. Meanwhile, the volume and content of negative electrolytes decrease, but the unavailable V^2+^ accumulates with cycling (Figure 1e). Hence, the capacity of the VRFB can be improved by oxidizing the unavailable V^2+^ to V^3+^ with air, and the working process is depicted in Figure 1e.

### Oxidization of V^2+^ with Air Affects VRFB

Nevertheless, the oxidation of V^2+^ with air is usually regarded as a side reaction.^5,27^ Therefore, we comprehensively analyzed the accumulation process of V^2+^ on the negative side during cycling and investigated the effects of oxidizing the negative electrolytes with air on the capacity of VRFBs. First, we analyzed all the vanadium ion crossover effects on the ion evolution in the charge/discharge process separately, as shown in Figure S3. Figure S3a shows that, although the crossover of V^2+^ and V^3+^ consumes different amounts of VO_2_^+^on the positive side, both finally result in the same amount of unavailable V^2+^ accumulation on the negative side. Similarly, the crossover of VO^2+^ and VO_2_^+^ consumes different amounts of V^2+^ on the negative side, and both finally result in the same amount of unavailable VO_2_^+^ accumulation on the positive side, as shown in Figure S3b. This means that the accumulated amount of unavailable V^2+^ is constant at a certain net flux of electrolyte, no matter how much the vanadium ions (V^2+^, V^3+^, VO^2+^, VO_2_^+^) contribute to the crossover separately. Moreover, the concentration and volume of the electrolyte increase on the positive side and decrease on the negative side with cycling^7^ due to the much higher diffusion rate across the Nafion series membranes of V^2+^ in comparison to other vanadium ions.^3^ The electrolyte change during VRFB cycling results in a surplus of V^2+^ on the negative side.^4,5^ Therefore, we used the net flux of V^2+^ (the amount of vanadium ion (V^2+^/ V^3+^) crossover from the negative to the positive sides subtracted by the amount of vanadium ion (VO^2+^/VO_2_^+^) crossover from the positive to the negative sides) to depict the effects of vanadium ion crossover on the ion evolution in the charge/discharge process for brevity.

**Figure 2:**
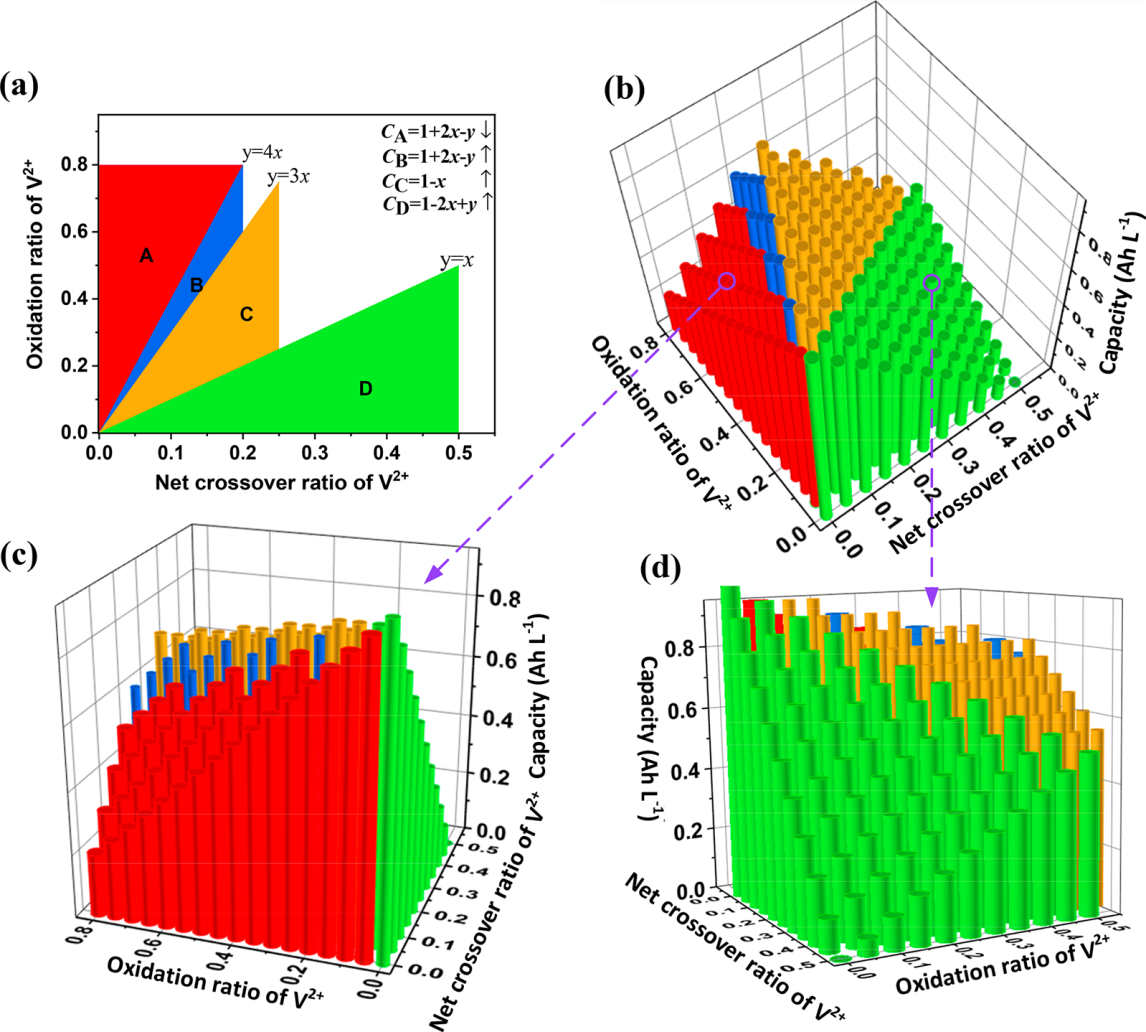
Effects of crossover and oxidation amounts of V^2+^ with air on the discharge capacity of VRFBs. A 2D floor plan (a) and 3D stereogram (b) of the oxidation effect of V^2+^ on the discharge capacity. Partial view of regions with negative (c) and positive (d) effects of oxidizing V^2+^ on the discharge capacity (*C*_A_, *C*_B_, *C*_C_, and *C*_D_ denote the capacity of the VRFB in different areas).

Herein, the initial capacity of VRFB is defined as 1; *x* (0 < *x* < 0.5 due to the two consumptions of VO_2_^+^) is the net flux ratio of V^2+^ transport from the negative to the positive side by crossover, and *y* is the oxidation ratio of V^2+^ with air. Thus, the oxidation ratio of V^2+^ with air (*y*) effects on the capacity of VRFBs under different crossover conditions (*x*) can be deduced (a detailed analysis can be found in the Supporting Information), as shown in Figure 2.

**Figure 3:**
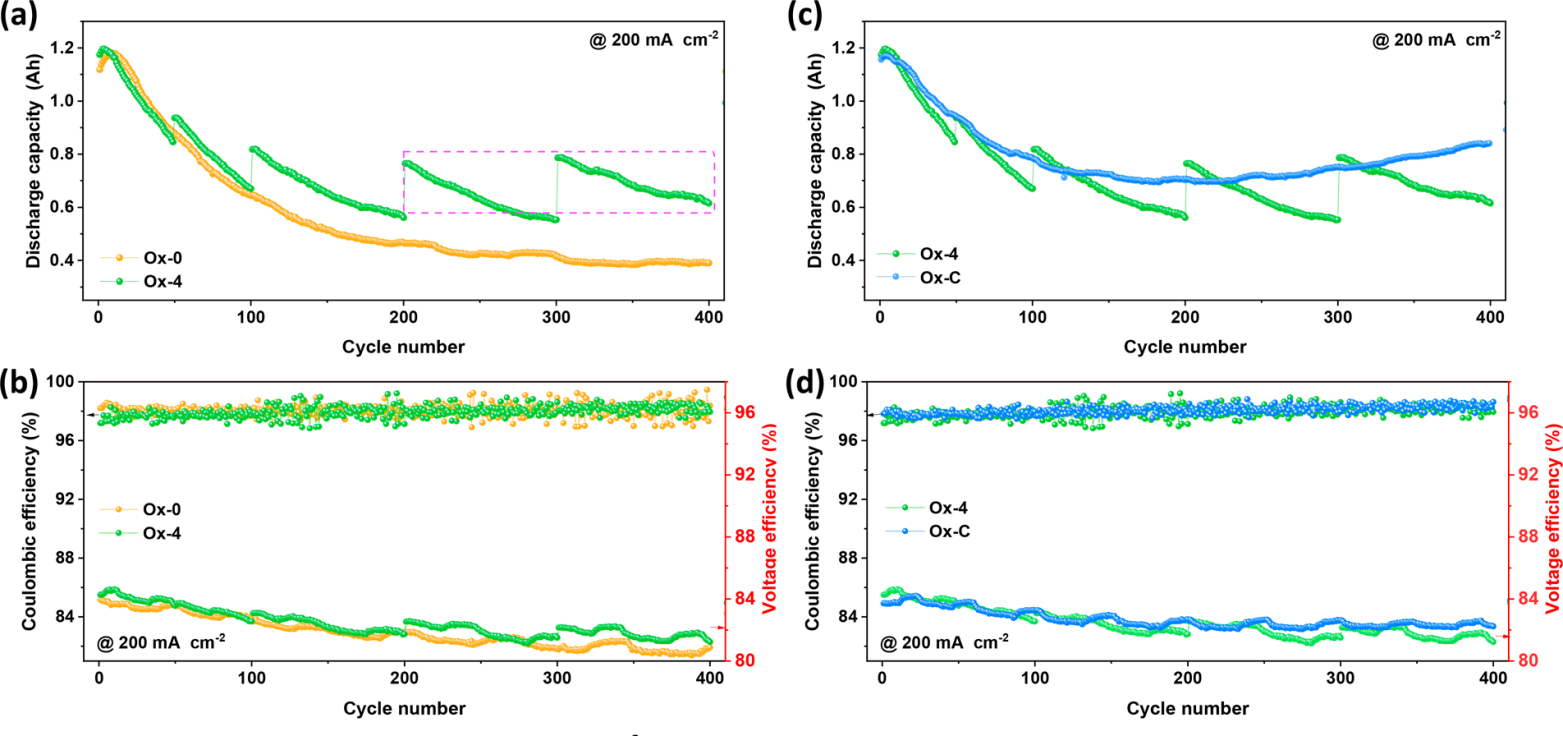
Intermittent oxidation of V^2+^ with air effects on (a) discharge capacity and (b) Coulombic efficiency and voltage efficiency of VRFBs. (c) Continued oxidation of V^2+^ with air effects on discharge capacity and (d) Coulombic efficiency and voltage efficiency of VRFBs.

Figure 2a,c shows that the oxidation of V^2+^ with air decreases the capacity of VRFBs at the initial stage (area A), which is consistent with the current popular perception.^7,9^ However, no research has yet revealed that the oxidation of V^2+^ with air can restore the capacity to different degrees in areas B–D of Figure 2a,b, especially when the amounts of crossover and oxidation both have large values (Figure 2d). To further examine the specific effect of oxidizing V^2+^ with air on the capacity of VRFB, VRFBs with the commercial electrolyte (V^3.50+^) are tested with and without air oxidation during cycling, as shown in Figure 3.

In Figure 3, Ox-0 presents the VRFB operated with nitrogen protection without V^2+^ oxidized by air during 400 cycles. Ox-4 denotes a VRFB with 4 times of air oxidation of V^2+^ at the 50th, 100th, 200th, and 300th cycles during cycling (detailed operations can be found in Figure S2a). Noticeably, a gradient of decreasing current density (312.5–200–125–62.5–50–18.75 mA cm^–2^) was applied before oxidizing the negative electrolyte with air to ensure the accumulated unavailable V^2+^ is oxidized completely without excess oxidation (on the line of *y* = *x* in Figure 2a). As shown in Figure 3a, compared with Ox-0, the discharge capacity with Ox-4 increases significantly after oxidizing with air at the 300th cycle, but the increment decreases with a decrease in cycle number. The reason is that, in cycles 10–200, the capacity decay of VRFB is mainly caused by the decrease of active species on the negative side.^16^ However in cycles 200–400, it is caused by the valence unbalance of the electrolytes on the positive and negative sides because the discharge capacity can be fully recovered after oxidizing the negative electrolyte with air at the 300th cycle compared with that at the 200th cycle. Thus, the different capacity decay mechanisms of VRFBs at different stages result in the increment of V^3+^ at 200th and 300th cycles being larger than that at the 50th and 100th cycles after oxidizing the negative electrolyte with air. Additionally, the larger increment of VE at the 200th and 300th cycles (Figure 3b) further boosts their capacity improvements under the same operating voltage range (0.9–1.6 V) during the round-trip charge/discharge process. Furthermore, Figure 3b shows that both Ox-0 and Ox-4 present excellent stability for ion selectivity during cycling, suggesting that the oxidation of V^2+^ with air does not influence the membrane’s ion selectivity.

On the other hand, since the capacity decay in cycles 200–300 is caused by a valence unbalance (the accumulation of unavailable V^2+^), the discharge capacity of VRFBs will keep stable during cycles 200–300 if we oxidize the unavailable V^2+^ with air in a timely and continual manner. Therefore, we tested the VRFB (swnoted Ox-C) performance with continual air oxidation of negative electrolyte during cycling (detailed operations can be found in Figure S2b)), as shown in Figure 3c,d. As expected, Ox-C displays a relatively stable discharge capacity during cycles 200–300. Furthermore, the discharge capacity of Ox-C in cycles 200–300 is close to the initial value of Ox-4 at cycles 200 and 300, further confirming that the capacity decay of Ox-0 after 200 cycles is caused by the nonequilibrium valence. Compared with Ox-0, the accumulated discharge capacity of Ox-C in 400 cycles is improved by 23.64%, increasing from 256.97 to 317.73 Ah. Additionally, the discharge capacity of Ox-C rises after 300 cycles, while the corresponding VE and CE do not increase in this range (Figure 3d). This is caused by the increasing osmotic pressure hydraulic gradient between the two sides of the membrane (Figure S7) after 300 cycles driving the net flux of the active species transport from the positive to the negative side and diminishing lack of V^3+^ in the anolyte.

### Elevating the Valence of Electrolytes Affects Battery Performance

**Figure 4:**
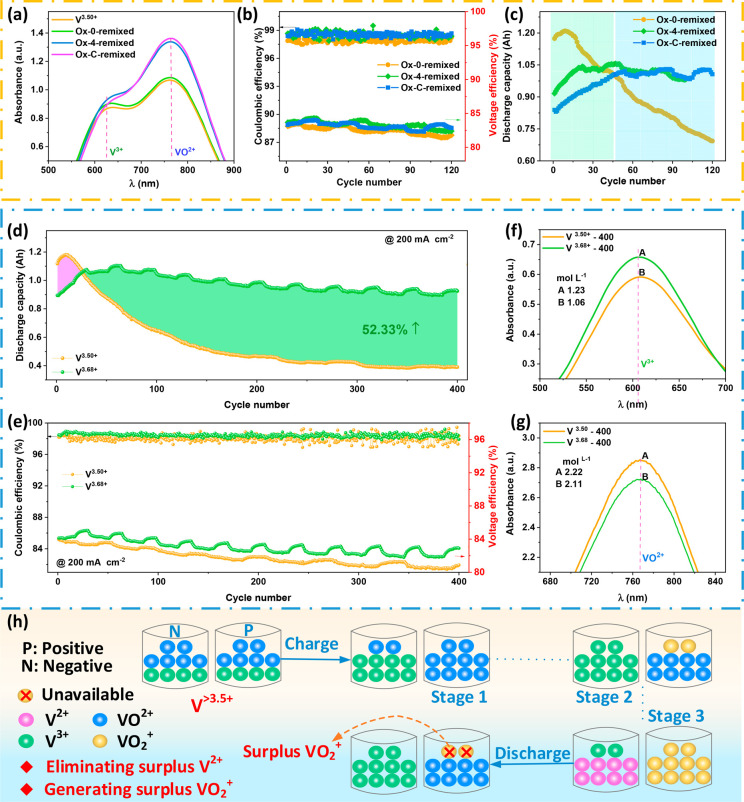
(a) UV spectra of the different electrolytes. (b) Coulombic efficiency and voltage efficiency and (c) discharge capacity of VRFBs with remixed electrolyte for Ox-0, Ox-4, and Ox-C after 400 cycles. (d) Coulombic efficiency and voltage efficiency and (d) discharge capacity of VRFBs with V^3.50+^ and V^3.68+^ electrolytes. (f, g) UV spectra of the V^3.68+^ and V^3.50+^ electrolytes after 400 cycles. (h) Schematic diagram of vanadium ion evolution of the VRFB with elevated valence electrolytes (V^>3.5+^) in the charge/discharge process.

In fact, the average valence of electrolytes will increase to some extent after oxidizing the negative electrolytes with air, which is validated by the UV results after remixing the electrolytes on the positive and negative sides, as shown in Figure 4a. In Figure 4, Ox-0-remixed, Ox-4-remixed, and Ox-C-remixed present the electrolytes remixing the positive and negative electrolytes of Ox-0, Ox-4, and Ox-C after 400 cycles, respectively (detailed operations can be found in Figure S1), and V^3.50+^ is the balance valence of the fresh commercial electrolyte. Figure 4a shows that the average valences of the remixed electrolytes for Ox-4 and Ox-C are obviously higher than that of the balanced valence of V^3.50+^, while that of Ox-0-remixed is very close to V^3.50+^. Moreover, elevating the average valence of electrolytes will decrease the maximum capacity of a VRFB,^28^ and this is also reflected in the VRFBs with the remixed electrolytes of Ox-4 and Ox-C, as shown in Figure 4c.

Figure 4c demonstrates that the discharge capacity of Ox-C-remixed at the initial stage is smaller than that of Ox-4-remixed, and both are lower than that of Ox-0-remixed due to the electrolytes’ average valence being elevated to different degrees (Figure 4a). However, the discharge capacity of Ox-4-remixed and Ox-C-remixed first increases rapidly with cycling and then keeps stable at the same value. Meanwhile, the discharge capacity of Ox-0-remixed presents the same trend as the fresh electrolyte (Figures 2 and 3): i.e., increasing temporarily and then decreasing rapidly. The elevated average valence of Ox-4-remixed and Ox-C-remixed enables them to present a better balance of electrolytes than Ox-0-remixed, which is also demonstrated in the increasing CE (Figure 4b). Moreover, the similar VEs of Ox-0-remixed, Ox-4-remixed, and Ox-C-remixed signify that the discrepancy of the electrolyte average valence mainly influences the vanadium ion transport across membranes (Figure 4h) and leads to different capacity trends with cycling. The schematic of the vanadium ion evolution in the charge/discharge process with and without oxidation is demonstrated in Figure S8.

As was mentioned above, the crossover of V^2+^ results in the lack of VO_2_^+^ on the positive side and the accumulation of unavailable V^2+^ on the negative side, which significantly aggravates the capacity decay. The VRFB with an elevated valence electrolyte generates surplus VO_2_^+^ after a charge/discharge cycle (Figure 4h). The available content of VO^2+^ increases after V^2+^ crossover from the negative to positive sides and reacts with the surplus VO_2_^+^, which contributes to the capacity increase in the first 35 cycles. Moreover, the surplus VO_2_^+^ eliminates the accumulation of V^2+^, reduces the crossover of V^2+^ from the negative to the positive sides, and enables VRFBs to present a high capacity retention rate in cycling. Furthermore, the decreased crossover of V^2+^ reduces the requirement of convection effects at the steady stage,^18^ which leads to a dynamic balance between diffusion and convection for Ox-4-remixed and Ox-C-remixed (about 35 cycles) arriving earlier than that of Ox-0 and the fresh electrolyte (about 200 cycles (Figure 2a). Hence, Ox-4-remixed and Ox-C-remixed present a larger capacity at the stable stage than that of Ox-0, and this is also reflected in the concentrations of V^3+^ and VO^2+^ after cycles (Figure S9).

Inspired by the stable performance of Ox-4-remixed and Ox-C-remixed, we first proposed a strategy to suppress the capacity decay of VRFBs by elevating the average valence of electrolytes. We prepared a fresh electrolyte with an elevated average valence according to the remixed electrolyte of Ox-4 after 400 cycles due to its good performance (Figure 4c). Specifically, the valence state of the remixed electrolyte of Ox-4 after 400 cycles is 3.68 (Figure S10). Next, the battery performance of a VRFB with the prepared elevated average valence electrolyte (denoted V^3.68+^) is compared with the commercial balanced valence electrolyte (denoted V^3.50+^) in Figure 4d,e.

Figure 4d shows that the discharge capacity decay of VRFB with V^3.68+^ is successfully suppressed compared with that of V^3.50+^, and it presents the same trend as that with Ox-4-remixed (Figure 4c). Noticeably, the accumulated discharge capacity in 400 cycles of the VRFB with V^3.68+^ is improved by 52.33% from 256.97 to 391.43 Ah than that with the commercial balanced valence electrolyte (V^3.50+^). Meanwhile, benefiting from the smaller concentration loss, the VE of VRFB with V^3.68+^ is also remarkably improved over that of V^3.50+^ (Figure 4e). Furthermore, the difference between V^3+^ and VO^2+^ concentrations of the VRFB with V^3.68+^ after 400 cycles is significantly smaller than that of V^3.50+^ (Figure 4f,g) under similar volume changes (Figure S11), implying that elevating the valence of electrolytes can effectively suppress the vanadium ion crossover and improve capacity retention. Besides, the discharge capacity and voltage efficiency of the VRFB coupled with V^3.68+^ present a larger fluctuation frequency with cycling than that of the VRFB coupled with V^3.50+^, but they display a similar fluctuation amplitude. The reason for the fluctuation is the varying room temperature, which changes regularly with time in our laboratory. However, the discharge capacity of the VRFB coupled with V^3.50+^ is much smaller than that with V^3.68+^ after 200 cycles (Figure 4d), leading to a much smaller fluctuation frequency with cycles for the former. Moreover, the rapid capacity decay of the VRFB with V^3.50+^ in the first 150 cycles also concealed the fluctuation phenomenon.

## Conclusions

We investigated the unavailable V^2+^ accumulating on the negative side with cycling caused by the crossover and found that the surplus V^2+^ greatly decreases the discharge capacity of VRFBs. Furthermore, we oxidized the unavailable V^2+^ to V^3+^ with air with intermittent and continuous strategies, significantly improving the discharge capacity. Unavoidably, the oxidation of V^2+^ with air will elevate the average valence of the remixing electrolytes after long-term cycling and lead to a relatively lower discharge capacity of VRFBs with the remixed electrolytes at the first few cycles compared with one without oxidation. Surprisingly, the VRFBs with an elevated valence of electrolytes present an excellent capacity retention rate in long-term cycling by generating surplus VO_2_^+^ on the positive side after a charge/discharge cycle. The surplus VO_2_^+^ on the positive side eliminates the accumulation of unavailable V^2+^ on the negative side, which reduces the transport of V^2+^ from the negative to the positive side. Furthermore, eliminating the accumulation of unavailable V^2+^ leads to the stable stage arriving earlier and lasting longer. Inspired by this, we propose a simple, facile, yet effective strategy to suppress the capacity decay of VRFBs by elevating the average valence of electrolytes. We designed a fresh electrolyte with an elevated average valence of V^3.68+^ to suppress the capacity decay of VRFBs. The results show that the accumulated discharge capacity in 400 cycles of VRFB with V^3.68+^ is greatly improved by 52.33% compared with the commercial electrolyte (V^3.50+^). This work provides deep insights into the capacity decay mechanism of VRFBs and develops an efficient and easily scaled-up electrolyte design method for suppressing the capacity decay of VRFBs, showing great potential for engineering applications in the near future.

## Methods

No unexpected or unusually high safety hazards were encountered. Details of experiments and the deduction process of crossover effects on vanadium ion concentration changes are described in Supporting Information.

## Supporting Information Available

Details regarding the experiments, UV spectrophotometry, electrolyte preparation, remixing electrolytes, viscosity measurements, battery tests, oxidizing V^2+^ with air, materials used in this work, the deduction process of crossover and oxidizing V^2+^ with air effect the on capacity of VRFBs (PDF)
Transparent Peer Review report available (PDF)
