# Supplementary material for: An Electrolyte with Elevated Average Valence for Suppressing the Capacity Decay of Vanadium Redox Flow Batteries
Source: ACS Cent Sci. 2022 Dec 23;9(1):56–63. doi: 10.1021/acscentsci.2c01112 (PMC9881198; doi:10.1021/acscentsci.2c01112)
Supplement: Supplementary file 1 — oc2c01112_si_001.pdf [file oc2c01112_si_001.pdf]

An Electrolyte with Elevated Average Valence for Suppressing the Capacity Decay of Vanadium Redox Flow Batteries

Zhenyu Wang[a]†, Zixiao Guo[a]†, Jiayou Ren[a], Yiju Li[b], Bin Liu[a], Xinzhuang Fan[a,], Tianshou Zhao[a,b,*]*

[a] Department of Mechanical and Aerospace Engineering, The Hong Kong University of Science and Technology, Clear Water Bay, Kowloon, Hong Kong, China

[b] Department of Mechanical and Energy Engineering, Southern University of Science and Technology, Shenzhen, 518055, China

†These authors contribute equally

**Correspondence: Xinzhuang Fan, mexzfan@ust.hk (X.Z. Fan); Tianshou Zhao, zhaots@sustech.edu.cn (T.S. Zhao).*

1. Experimental

1.1 Materials

In this work, GF with a thickness of 0.25 cm provided by Liaoning Jingu Carbon Material Co., Ltd was used as the electrodes of VRFB. The Nafion 212 bought from DuPon was used as the membrane of the VRFB. The electrolyte on the positive and negative sides was 1.70 M $V^{3.50+}$ /3.00 M H_2SO_4 supplied by Sichuan Xingming Energy Material Co., Ltd. All these electrodes, membranes, and electrolytes were used without any further treatment.

1.2 UV-vis spectrophotometry

The concentration of vanadium ions was measured with Lambda 365 UV-Visible Spectrophotometer (PerkinElmer, USA). In order to ensure VO_2^+ is consumed entirely after discharge, a gradient decreasing current density (312.5-200-125-62.5-50-18.75 mA cm^{-2}) is applied before testing the concentrations of vanadium ions.

1.3 Electrolyte preparation

The 1.70 M VO^{2+}/ 3.00 M H_2SO_4 electrolyte was obtained by charging the commercial electrolyte (1.70 M $V^{3.50+}$ /3.00 M H_2SO_4). The electrolyte with the valence of $V^{3.68+}$ was obtained by remixing 52.0 mL commercial electrolyte ($V^{3.50+}$) with 28.0 mL VO^{2+} electrolyte, and the actual valence state of the electrolyte is $V^{3.675+}$. In this work, we noted $V^{3.675+}$ as $V^{3.68+}$ to keep the form consistent with $V^{3.50+}$.

1.4 Remixing electrolytes

Before remixing the electrolytes on the positive and negative sides, a gradient decreasing current density (312.5-200-125-62.5-50-18.75 mA cm^2) is conducted to discharge the VRFBs to 0 SOC (stopped at 0 V). Next, inverting the pump speed to pump electrolytes into tanks; third, connecting the catholyte outlet to the anolyte outlet and pumping all the catholyte to the anolyte, and remixing the electrolytes. Finally, shake well the remixed electrolytes and divide the electrolytes equally after waiting for 1 h. The remixing electrolytes process is depicted in Fig. S1.

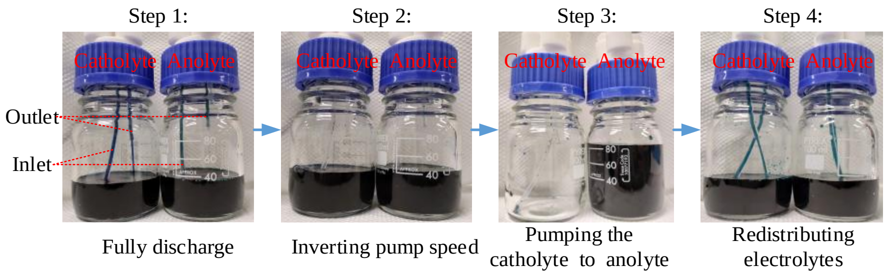

Fig. S1. Remixing electrolytes procedures.

1.5 Viscosity measurement

The viscosity of vanadium electrolytes is tested with the 1834 Ubbelohde viscometer in a thermostat HS0100 (Lorderan, China).

1.6 Battery tests

VRFBs were assembled with the zero-gap structure,[1] and the graphite plates with a serpentine flow field (SFF) were used as the flow field plate. Two pieces of GF with the size of 4.00 × 4.00 cm^2 were used as the electrodes on the positive and negative sides. 40.00 mL electrolytes were stored in the positive and negative tanks and pumped into the battery by a peristaltic pump Longer BT100 (Longer Precision Pump Co., Ltd, UK) with a flow rate of 2.50 mL min^{-1} cm^{-2}. The battery performances were recorded by Arbin 179539 (Arbin instruments, USA), and the operating voltage range was 0.90-1.60 V for all battery tests. During the tests, nitrogen was purged into the tank to avoid the oxidization of V^{2+} by air.

In this work, Ox-0 presents the VRFB operated with nitrogen protection without V^{2+} oxidized by air during 400 cycles. Ox-4 means the VRFB with 4 times air oxidization of V^{2+} at the 50th, 100th, 200th, and 300th cycles during cycling. Ox-C represents the VRFB that the negative electrolyte is oxidized continually with air during cycling. Ox-0-remixed, Ox-4-remixed, and Ox-C-remixed represent the VRFBs of Ox-0, Ox-4, and Ox-C with the remixed electrolytes after 400 cycles, separately. $V^{3.50+}$ and $V^{3.68+}$mean the VRFB coupled with the electrolyte of $V^{3.50+}$ and $V^{3.68+}$ separately. The detailed experimental setting parameters are listed in Tab. S1.

Tab. S1 The setting parameters in VRFB tests.

Batteries	Catholyte	Anolyte	Operating voltage range	Current density (mA cm^{-2})
Ox-0	40 mL 1.7 M $V^{3.50+}$	40 mL 1.7 M $V^{3.50+}$	1.6-0.9 V	200
Ox-4	40 mL 1.7 M $V^{3.50+}$	40 mL 1.7 M $V^{3.50+}$	1.6-0.9 V	200
Ox-C	40 mL 1.7 M $V^{3.50+}$	40 mL 1.7 M $V^{3.50+}$	1.6-0.9 V	200
Ox-0-remixed	Ox-0 after 400 cycles	Ox-0 after 400 cycles	1.6-0.9 V	200
Ox-4-remixed	Ox-4 after 400 cycles	Ox-4 after 400 cycles	1.6-0.9 V	200
Ox-C-remixed	Ox-C after 400 cycles	Ox-C after 400 cycles	1.6-0.9 V	200
$V^{3.50+}$	40 mL 1.7 M $V^{3.50+}$	40 mL 1.7 M $V^{3.50+}$	1.6-0.9 V	200
$V^{3.68+}$	40 mL 1.7 M $V^{3.68+}$	40 mL 1.7 M $V^{3.68+}$	1.6-0.9 V	200
Fully discharge	----	----	0 V	312.5-200-125-62.5-50-18.75

1.7 Oxidizing V^{2+} with air

This work used two different ways to oxidize V^{2+} with air. One is oxidizing V^{2+} after a full discharge (Ox-4), and the other is oxidizing V^{2+} with air continually during the charge/discharge cycling (OX-C). In the first method, a gradient decreasing current density (312.5-200-125-62.5-50-18.75 mA cm^{-2}) is applied to discharge the VRFBs to 0 SOC and avoid excessive oxidization of V^{2+}. Then, blow air into the negative electrolyte tank and ensure the pipe is inserted into electrolytes (Fig. S2(a)) to accelerate the oxidization process and ensure V^{2+} is oxidized completely. Moreover, a sealing film (PARAFILM@, USA) is used to avoid the electrolyte splash. This process continues 6 hours to ensure the oxidization of V^{2+} with air completely. For the continued oxidization of V^{2+} with air, we just loosen the negative tank's sealing cap and allow air exchanges slowly inside and outside the tank after the oxygen in the tank is consumed by V^{2+}, as shown in Fig. S2(b).

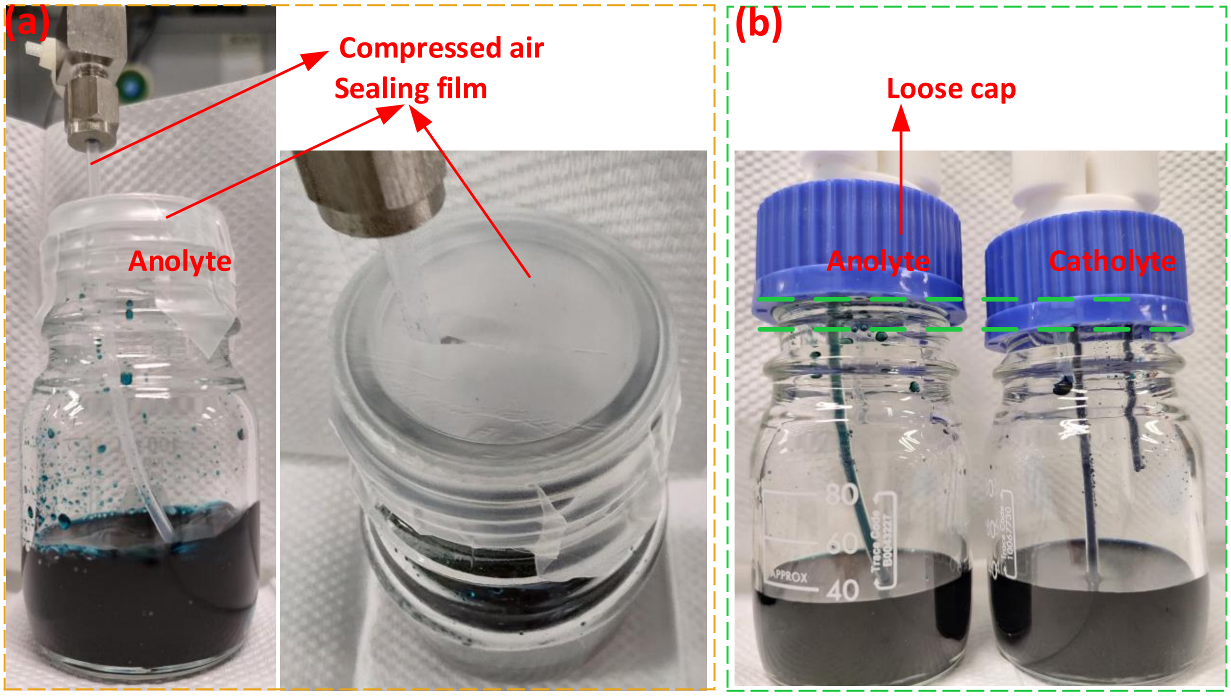

Fig. S2 Oxidization method of the unavailable V^{2+} in the anolyte. (a) Oxidizing V^{2+} with air at the end of a full discharge. (b) Oxidizing V^{2+} with air continually in the charge/discharge process.

2. Standby analysis

2.1The accumulation process of V^{2+} caused by crossover

In VRFBs, the side reactions (Eq. S1-Eq. S6)[2] will occur when vanadium ions cross the membrane from one side to the other side. The effect of crossover on vanadium ions' evolution in the charge/discharge process is depicted in Fig. S3. Fig. S3(a) shows that although the crossover of V^{2+} and V^{3+} consumes different amounts of VO_2^+ on the positive side, and results in the same amount of unavailable V^{2+} on the negative side finally. Similarly, the crossover of VO^{2+} and VO_2^+ consumes different amounts of V^{2+} on the negative side, and results in the same amount of unavailable VO_2^+ on the positive side finally, as shown in Fig. S3(b). That means the accumulated amount of unavailable V^{2+} is a constant at a certain net flux of the electrolyte, no matter how much the vanadium ions (V^{2+}, V^{3+}, VO^{2+}, VO_2^+) contribute to the crossover separately. Moreover, the

concentration and volume of the electrolyte increase on the positive side and decrease on the negative side with cycling[3] due to the much higher diffusion rate across the Nafion series membranes of V^{2+} than other vanadium ions (Tab. S2).[2] The electrolytes' changing results in the surplus of V^{2+} on the negative side.[4,5] Therefore, we used the net flux of V^{2+} to present the amount of vanadium ions (V^{2+}/ V^{3+}) crossover from the negative to the positive sides subtracting the amount of vanadium ions (VO^{2+}/VO_2^+) crossover from the positive to the negative sides. Then, we used the net flux of V^{2+} to depict the effects of vanadium ions crossover on the accumulation process of unavailable V^{2+} in the following calculation for brevity.

$$V^{2+} + 2VO_2^+ + 2H^+ \rightarrow 3VO^{2+} + H_2O \tag{S1}$$

$$V^{2+} + VO^{2+} + 2H^+ \rightarrow 2V^{3+} + H_2O \tag{S2}$$

$$V^{3+} + VO_2^+ \rightarrow 2VO^{2+} \tag{S3}$$

Side reactions for the vanadium ions transport from the positive to the negative sides:

$$VO_2^+ + 2V^{2+} + 2H^+ \rightarrow 3VO^{2+} + H_2O \tag{S4}$$

$$VO_2^+ + V^{3+} \rightarrow 2VO^{2+} \tag{S5}$$

$$VO^{2+} + V^{2+} + 2H^+ \rightarrow 2V^{3+} + H_2O \tag{S6}$$

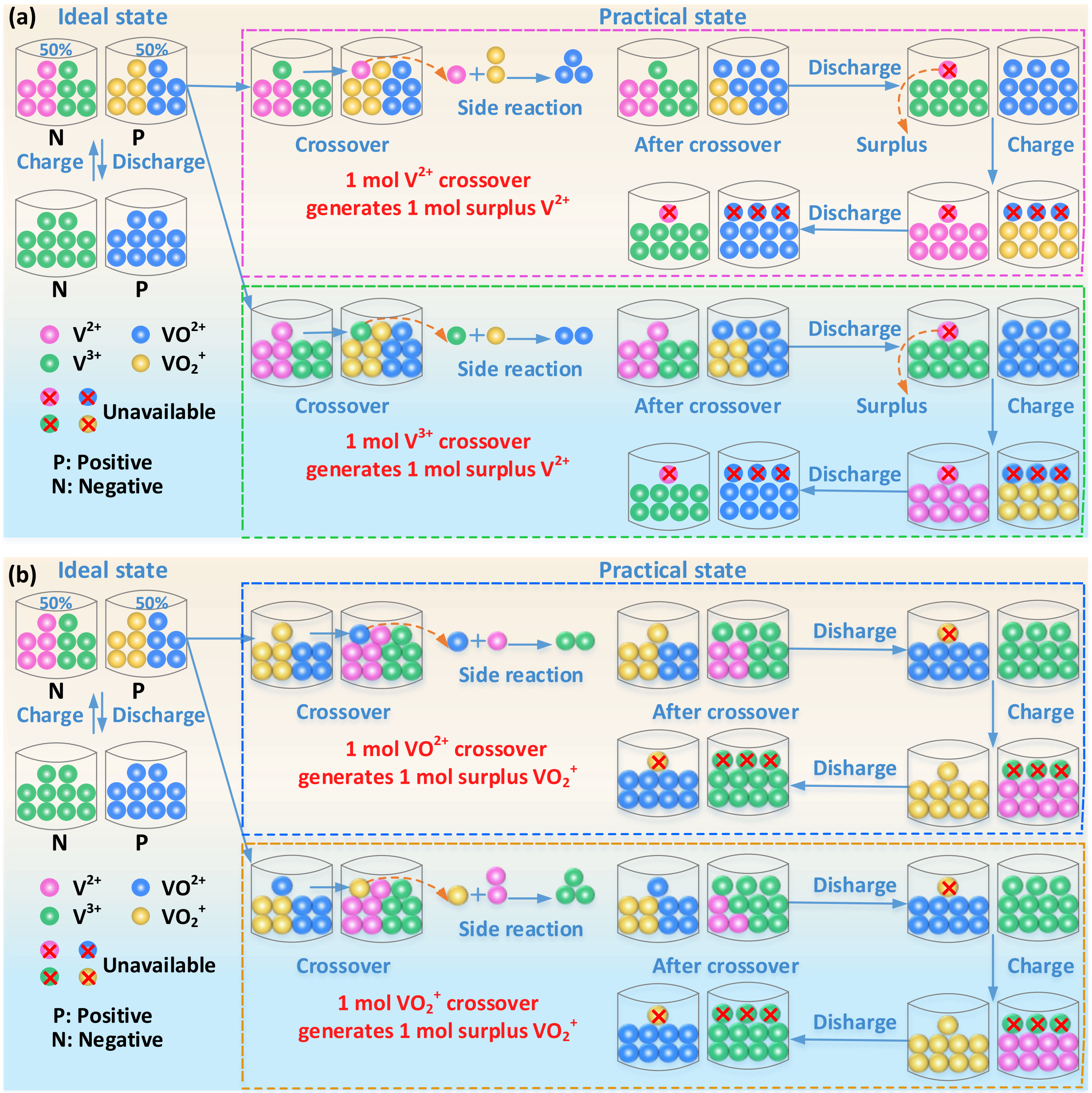

Fig. S3 Schematic of (a) V^{2+}/V^{3+} and (b) VO^{2+} /VO_2^+ crossover effect on the ions' evolution in the charge/discharge process.

Tab. S2 The diffusion rate of vanadium ions across Nafion 115.[3]

Vanadium ions	V^{2+}	V^{3+}	VO^{2+}	VO_2^+
Diffusion coefficient/$\times 10^{-6}$ cm^2 min^{-1}	5.261	1.933	4.095	3.538

2.2 The oxidization of V^{2+} with air affect the capacity of VRFB

Therefore, if we neglect the material degeneration, concentration, hydrogen, and oxygen evolution effects on the capacity decay, which is normally adopted in VRFB simulating studies,[6,7] the capacity decay imputes to the net flux of V^{2+} caused by crossover. Hence, if we define the electrolytes on the positive and negative sides, including 1 M VO_2^+ and V^{3+}separately at the initial stage; *x* (0<*x*<1) is the net flux of V^{2+} crossover from the negative side to the positive side after several cycles. Hence, based on Eq. (S1), the available capacity decay at different cycles can be written as Tab. S3.

Tab. S3 The variation of vanadium ions contents with crossover.

	Positive		Negative	
Vanadium ions	VO^{2+}	VO_2^+	V^{2+}	V^{3+}
Contents at the end of charge (M)	0	1	1	0
Contents at the end of discharge (M)	1	0	0	1
Available capacity (Ah L^{-1})	**1**×96485 /3600			
Net flux of V^{2+} after crossover (M)	*x*			
Contents at the end of charge (M)	3*x*	**1-2*x***	1-*x*	**0**
Contents at the end of discharge (M)	1+*x*	**0**	*x*	**1-2*x***
Available capacity (Ah L^{-1})	**(1-2*x*)** ×96485/3600			

*Where 96485 is the Faraday constant, 3600 is the seconds for 1 hour.

Tab. S3 shows that, essentially, the discharge capacity is limited by the contents of VO_2^+ at the end of the charge. The lack of VO_2^+ results in the deficiency of V^{3+} on the negative side in the discharge process, further limiting the generation of VO_2^+ in charge process. Thus, two ways can improve the discharge capacity: one is introducing additional VO_2^+ on the positive side, and the other is increasing the amount of V^{3+} on the negative side. Obviously, the second one presents an advantage due to reducing electrolyte consumption, and the redundant V^{2+} can easily be oxidized to V^{3+} in

the air. Therefore, we oxidized the unavailable V^{2+} with air during round-trip cycling and studied the effects on the capacity of VRFBs.

Before carrying out the oxidization method, the relationship between the amount of V^{2+} oxidization with air and the capacity of VRFB is analyzed. Based on Tab. S3, if we define *y* as the amount of V^{2+} that is oxidized with air in the round-trip cycling process, the variation of the capacity can be depicted in Tab. S4:

Tab. S4 The relationship between the oxidization amount of V^{2+} with air and the capacity of VRFB.

	Positive		Negative	
Vanadium ions	VO^{2+}	VO_2^+	V^{2+}	V^{3+}
Contents at end of charge (M)	0	1	1	0
Available capacity (Ah L^{-1})	**1**			
Net flux of V^{2+} after crossover (M)	***x***			
Contents at the end of charge (M)	3*x*	**1-2*x***	1-*x*	**0**
The amount of V^{2+} oxidized by air (M)	**y**			
Contents at end of charge (M)	3*x*	**1-2*x***	1-*x*-*y*	y
Available capacity (Ah L^{-1})	*f* (*x*, *y*)			

Tab. S4 shows that the capacity of VRFB is not simply dominated by the minimum contents of V^{2+} and VO_2^+ after the oxidizing of V^{2+} with air. The contents of VO^{2+} could also become the limit factor due to the excessive oxidization of V^{2+}. Thus, the possible contents of vanadium ions after oxidizing are listed in Tab. S5 at the end of discharge/charge.

Tab. S5 The contents of vanadium ions at the end of discharge after oxidization V^{2+} with air.

		Positive		Negative	
Vanadium ions		VO^{2+}	VO_2^+	V^{2+}	V^{3+}
Contents at end of charge (M)		3*x*	**1-2*x***	1-*x*-*y*	y
	Conditions				
Contents at end of discharge (M)	**1-*x*-*y*<1-2*x***	1+2*x*-*y*	*y*-*x*	0	1-*x*
	**1-*x*-*y*>1-2*x***	1+*x*	0	*x*-*y*	1-2*x*+*y*

Tab. S5 shows that the value of *x* and *y* dominate the capacity of VRFB. Therefore, we divided the capacity of VRFBs after oxidation with air into 4 parts as below:

$$\text{Aviliable capacity:}\begin{cases} 1-2x+y & (0 \le \mathrm{y} \le x < 0.5) & \uparrow \\ 1-x & (0 < x \le \mathrm{y} < 1-x)) \cap (3x > y) \cap (x < 0.5) & \uparrow \\ 1+2x-y & (0 < 3x < y < 1-x)) \cap (4x > y) \cap (x < 0.5) & \uparrow \\ 1+2x-y & (0 < 4x < y < 1-x)) \cap (x < 0.5) & \downarrow \end{cases} \quad \text{(S7)}$$

Compared with the available capacity (1-2*x*) of the VRFB after *x* M V^{2+} net flux transport from the negative to the positive sides, oxidizing V^{2+} with air can obviously improve the capacity of VRFB except when the oxidization amount of V^{2+} is 4 times larger than *x*.

3. Results

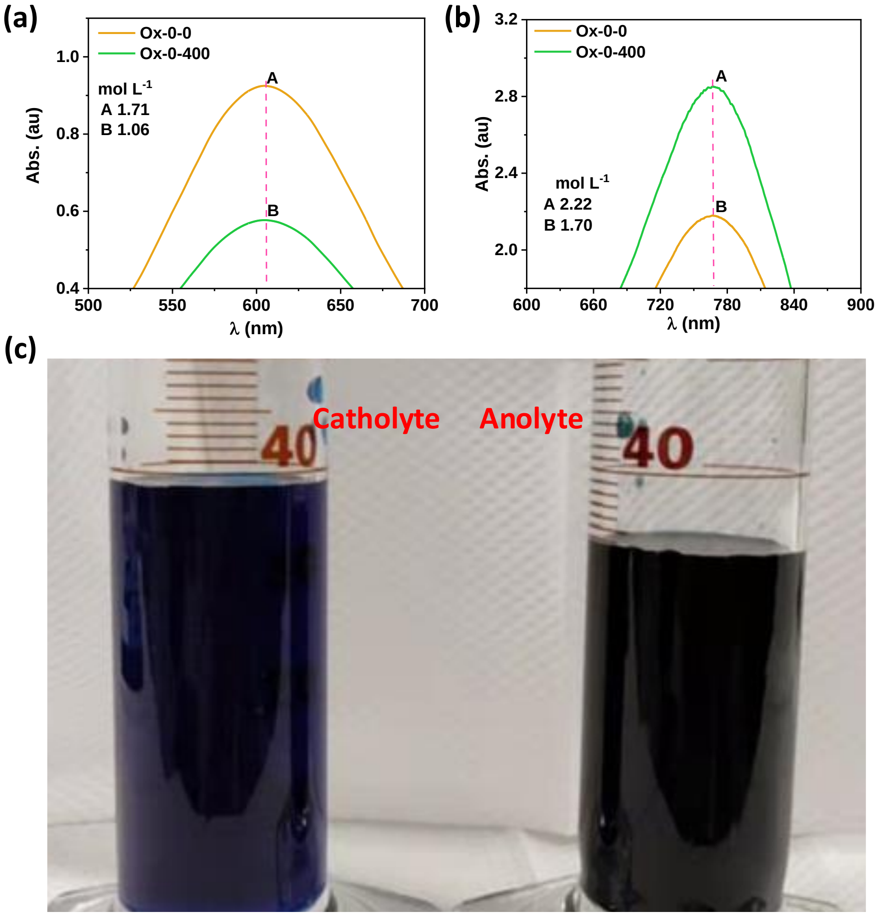

Fig. S4 The UV spectrums of (a) V^{3+}, (b) VO^{2+} and (c) the electrolytes' volume change in the VRFB coupled with $V^{3.50+}$ after 400 cycles.

In Fig. S4, Ox-0 represents the VRFB coupled with $V^{3.50+}$ operated with nitrogen protection during 400 cycles without V^{2+} oxidized by air. The Ox-0-0 and Ox-0-400 mean the electrolytes conditions at 0 cycle and after 400 cycling cycles.

Initially, 40 mL 1.71 M V^{3+} and 40 mL 1.70 M VO^{2+} were used as the anolyte and catholyte for VRFB respectively. Thus, the total vanadium ions can be calculated below.

$$40 \times 1.71 + 40 \times 1.70 = 136.4 \text{ mM} \quad \text{(S8)}$$

Suppose the total volume of the anolyte and catholyte in VRFBs is the same before and after cycling. However, the electrolytes cannot wholly be pumped out from the VRFB after cycling due to the electrode absorption and residual in pipes and channels, resulting in the total electrolytes after cycling obviously less than that at the initial stage, as shown in Fig. S4(c) (catholyte: 38.5 mL, anolyte: 34.8 mL). Next, suppose the electrolyte consumption (residual in the electrodes, pipes, and channels after pumping out the electrolytes) is the same on the positive and negative sides. Thus, the electrolyte consumption in each half cell can be calculated below.

$$\frac{1}{2}\left(\left(40+40\right)-\left(33.8+38.5\right)\right)=3.35\ \text{mL} \tag{S9}$$

Therefore, the actual volume of electrolytes on the negative and positive side of the VRFB after cycling can be obtained as below:

$$\text{Positive side:}\, 3.35+38.5=41.85\ \text{mL} \tag{S10}$$

$$\text{Negativ side:}\, 3.35+33.8=37.15\ \text{mL} \tag{S11}$$

Hence, the total vanadium ion in the electrolyte of VRFB after 400 cycles can be calculated as Eq. (S12). And the calculated result is very close to that at the initial stage.

$$37.15\times 1.06+41.85\times 2.22=132.286\ \text{mM} \tag{S12}$$

Due to the unavailable V^{2+} still existing on the negative side after full discharge, which readily reacts with air, a sealing gat is used during the UV test. The concentrations of V^{3+} tested with UV might be slightly higher than the actual value because it is unavoidable to expose the electrolyte in the air for a short time during electrolyte transformation.

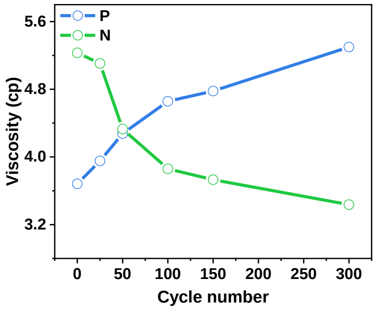

Fig. S5 The viscosity of the electrolytes in the VRFB coupled with $V^{3.5+}$ changes with cycling at 0 SOC (P: positive electrolyte, N: negative electrolyte).

Fig. S5 shows that the viscosity of electrolytes in the catholyte increases rapidly in the first 100 cycles and then slows down the increasing rate, which is consistent with the change in electrolytes' concentration with cycling. Similarly, the viscosity of electrolytes in the anolyte presents the same trend as that of the electrolytes' concentration change with cycling.

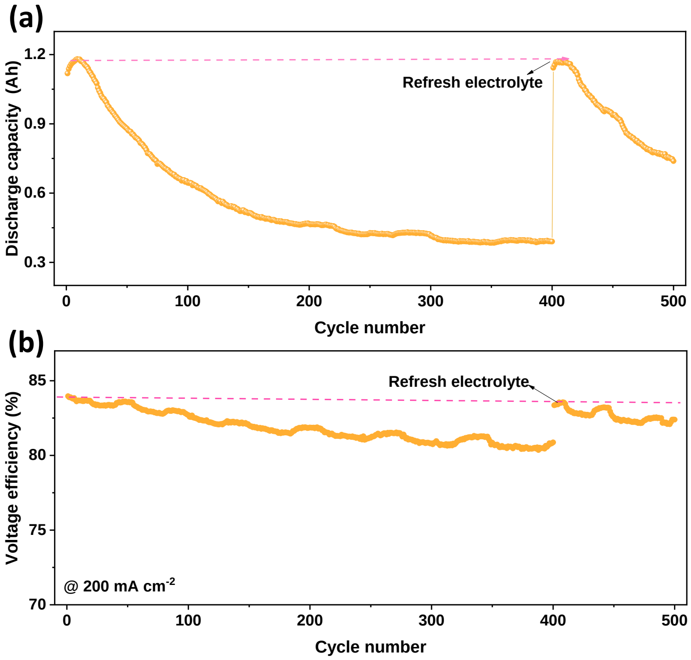

Fig. S6 The (a) capacity and (b) voltage efficiency of VRFB before and after refreshing the electrolyte.

Fig. S6 shows that both the discharge capacity and VE are recovered after refreshing the electrolyte, which means the capacity and VE decay in the first 400 cycles are caused by the changing of electrolytes.

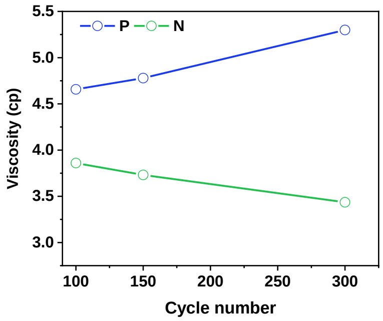

Fig. S7 The viscosity of OX-C changes with cycling at 0 SOC (P: positive electrolyte, N: negative electrolyte).

In Fig. S7, The Ox-C represents the VRFB that the negative electrolyte is continually oxidized with air during cycling. Fig. S7 shows that the gap of viscosity between the negative and positive electrolytes of Ox-C increases with cycling, which increase the amount of vanadium ions transported from the positive to the negative sides by convection and diminishes the lack of V^{3+} on the negative side. Thus, the capacity of Ox-C increases with the cycling after 300 cycles due to the increment of negative active species.

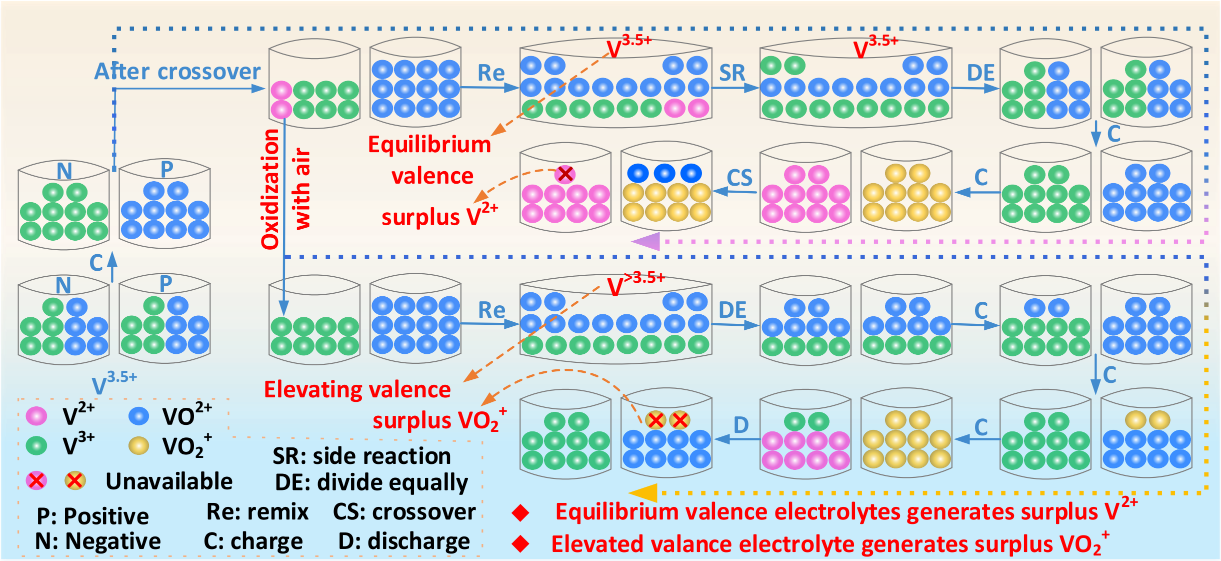

Fig. S8 The schematic of the vanadium ions evolution in the charge/discharge process of VRFBs with conventional remixed electrolytes and the oxidized electrolytes.

The concentrations of Ox-0-remixed, Ox-4-remixed, and Ox-C-remixed after 120 cycles are compared, as shown in Fig. S9.

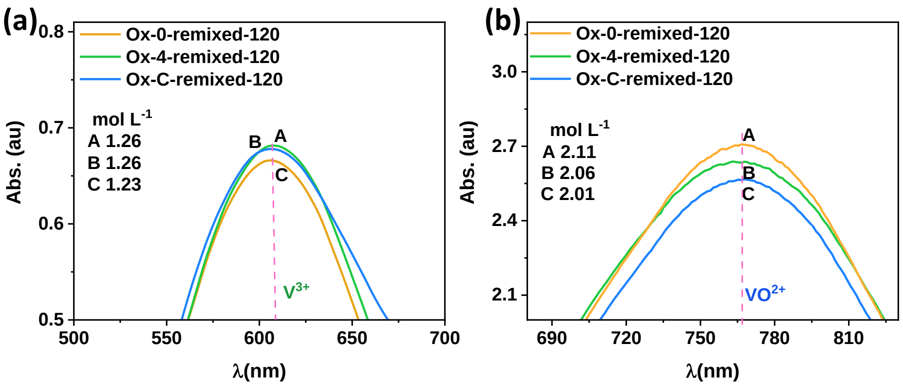

Fig. S9 The comparison of electrolytes' concentration of Ox-0-remixed, Ox-4-remixed, and Ox-C-remixed on the (a) negative and (b) positive sides after 120 cycles.

In Fig. S9, Ox-0-remixed, Ox-4-remixed, and Ox-C-remixed represent the VRFBs of Ox-0, Ox-4, and Ox-C with the remixed electrolytes after 400 cycles separately. Fig. S9 shows that the difference in electrolyte concentration on the positive and negative sides after 120 cycles were

narrowed with the Ox-4-remixed and Ox-C-remixed electrolytes compared with that of Ox-0-remixed, which benefits the capacity retention (Fig. 4(c)). That means the electrolyte with elevated valence diminishes the vanadium ions crossover and contributes to maintaining high capacity retention of VRFBs.

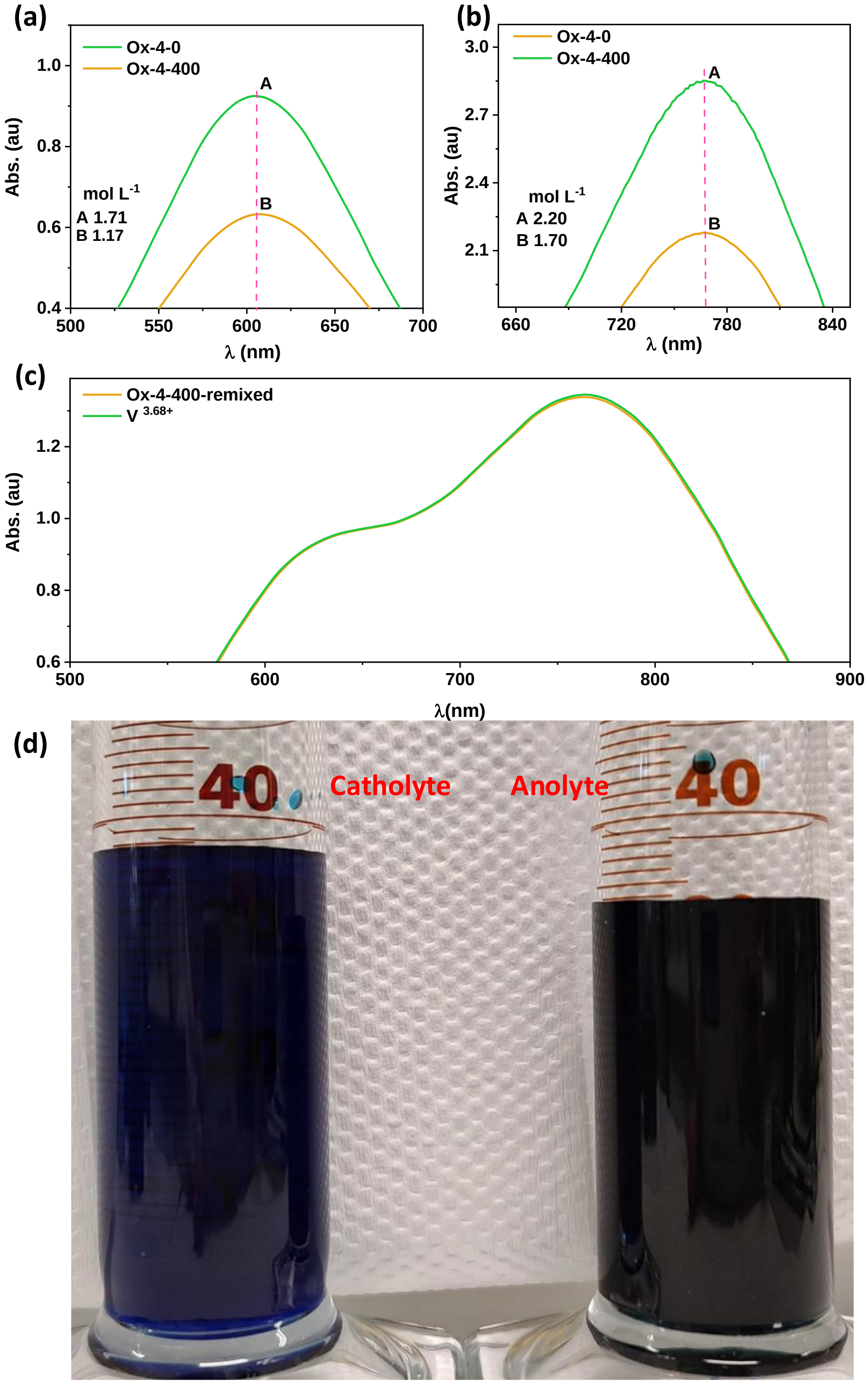

Fig. S10 The UV spectrum of Ox-4 electrolytes before and after 400 cycles on (a) the positive and (b) negative sides. (c) The UV spectrum comparison of prepared elevated valence electrolytes with the remixed electrolyte of Ox-4 after 400 cycles. (d) The electrolytes' volume of Ox-0 after 400 cycles.

The elevated valence of the electrolyte was prepared based on the concentration and volume changes of Ox-0-remixed after 400 cycles (Fig. S10). The electrolyte volume on the positive and negative sides is shown in Fig. S10(d). Suppose the consumption of the electrolyte (residual in the electrodes, pipes, and channels after pumping out the electrolytes) on the positive and negative sides are the same. Thus, the electrolyte consumption in half of the VRFB can be calculated as below:

$$\frac{1}{2}\left(\left(40+40\right)-\left(34.0+38.0\right)\right)=4.0\ \text{mL} \tag{S13}$$

Therefore, the real volume of the electrolytes on the negative and positive sides of the VRFB after cycling can be obtained as below:

$$\text{Positive side: } 4.0+38.0=42.0\ \text{mL} \tag{S14}$$

$$\text{Negativ side: } 4.0+34.0=38.0\ \text{mL} \tag{S15}$$

$$\left(42.0\times2.20\times4+38.0\times1.17\times3\right)\Big/\left(42.0\times2.20+38.0\times1.17\right)=3.675 \tag{S16}$$

Thus, the electrolytes with the valance of 3.675 (noted as $V^{3.675+}$) can be obtained by mixing the VO^{2+} (1.7 M VO^{2+}/ 3 M H_2SO_4) with the electrolytes of $V^{3.50+}$. If we defined x is the volume of the VO^{2+} that is used to prepare the electrolytes ($V^{3.675+}$) with 80 mL, the following relationship can be obtained:

$$\left(\left(80-x\right)\times3.5+4x\right)/80=3.675 \tag{S17}$$

By solving Eq. (S17), the proportion of $V^{3.50+}$ and VO^{2+} is obtained. The volumes of $V^{3.50+}$ and $VO^{2+}$are 52.0 mL and 28.0 mL, respectively. In this work, we noted $V^{3.675+}$ as $V^{3.68+}$ to keep the form consistent with $V^{3.50+}$.

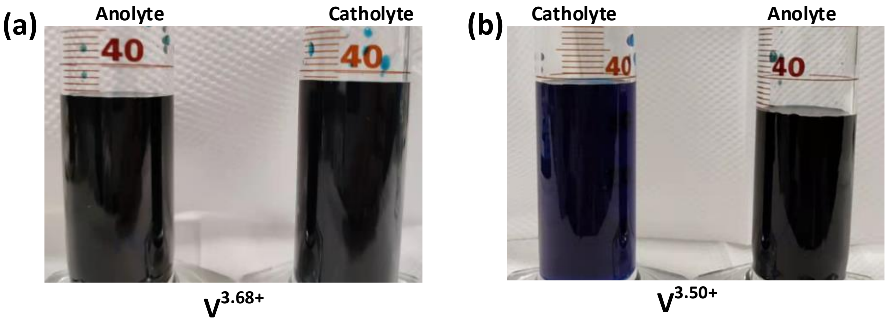

Fig. S11 The volume changes of electrolytes $V^{3.68+}$ and $V^{3.50+}$ after 400 cycles.

Fig. S11 shows that the electrolytes' volume of VRFB coupled with $V^{3.50+}$ and $V^{3.68+}$ are similar after 400 cycles.

References

(1) Wei L, Zhao T S, Zhao G, An L, and Zeng L. A high-performance carbon nanoparticle-decorated graphite felt electrode for vanadium redox flow batteries[J]. Applied energy, 2016, 176: 74-79. DOI: 10.1016/j.apenergy.2016.05.048

(2) Li Y, Skyllas-Kazacos M, Bao J. A dynamic plug flow reactor model for a vanadium redox flow battery cell[J]. Journal of Power Sources, 2016, 311: 57-67. DOI: 10.1016/j.jpowsour.2016.02.018

(3) Sun C, Chen J, Zhang H, Han X, and Luo Q. Investigations on transfer of water and vanadium ions across Nafion membrane in an operating vanadium redox flow battery[J]. Journal of Power Sources, 2010, 195(3): 890-897. DOI: 10.1016/j.jpowsour.2009.08.041

(4) Song Y, Li X, Xiong J, Yang L, Pan G, Yan C, and Tang A. Electrolyte transfer mechanism and optimization strategy for vanadium flow batteries adopting a Nafion membrane[J]. J Power Sources, 2020, 449: 227503. DOI: 10.1016/j.jpowsour.2019.227503

(5) Tang A, Bao J, Skyllas-Kazacos M. Dynamic modelling of the effects of ion diffusion and side reactions on the capacity loss for vanadium redox flow battery. *J Power Sources*. 2011;196(24):10737–47. DOI: 10.1016/j.jpowsour.2016.02.018

(6) Knehr K W, Agar E, Dennison C R, Kalidinadi A R, and Kumbur E C. A transient vanadium flow battery model incorporating vanadium crossover and water transport through the membrane[J]. Journal of The Electrochemical Society, 2012, 159(9): A1446. DOI:10.1149/2.017209jes

(7) Chen H, Cheng M, Feng X, et al. Analysis and optimization for multi-stack vanadium flow battery module incorporating electrode permeability[J]. Journal of Power Sources, 2021, 515: 230606. DOI: 10.1016/j.jpowsour.2021.230606
