# Supplementary material for: An Electrolyte with Elevated Average Valence for Suppressing the Capacity Decay of Vanadium Redox Flow Batteries
Source: ACS Cent Sci. 2022 Dec 23;9(1):56–63. doi: 10.1021/acscentsci.2c01112 (PMC9881198; doi:10.1021/acscentsci.2c01112)
Supplement: Supplementary file 2 — oc2c01112_si_002.pdf [file oc2c01112_si_002.pdf]

oc-2022-01112j.R1

Name: Peer Review Information for "An Electrolyte with Elevated Average Valence for Suppressing the Capacity Decay of Vanadium Redox Flow Batteries"

First Round of Reviewer Comments

Reviewer: 1

Comments to the Author

This manuscript investigated the capacity decay behavior of VFBs caused by the crossover of the V2+. In addition to the vanadium ion loss of the negative electrolytes, authors propose the unavailable V2+ arisen from the double consumption of VO2+ induces the capacity decay in the long-term cycling. Herein, the manuscript demonstrates a simple strategy to suppress the capacity fading by enhancing the average valance of the original electrolyte (V3.68+), indicating an improvement of 52.33% in the accumulated discharge capacity during 400 cycles of the VFB compared with the commercial electrolyte. The topic is interesting and paper is well organized. Therefore, I suggest publishing this paper after considering the following comments:

1. In the abstract and introduction, research on membranes was mentioned and reviewed. However, this work is about electrolyte modification instead of the design of membranes. Please quote related references and review the progress in this field.

2. In Figure 1c, based on the calculation, the concentration of the accumulated V2+ (0.05 M) seems too low to have a sever impact on the capacity retention according to the author’s point of view, that the accumulated V2+ greatly decreases the discharge capacity of VRFBs after long-term cycling. Please explain.

3. In Figure 1e, the schematic diagram vividly shows the formation of the unavailable V2+ and the capacity improvement through oxidation V2+ with air. However, no schematic diagram to illustrate the concept of the electrolyte with elevated average valence. If it is possible to add the schematic diagram of the evolution of vanadium ions during the charge/discharge processes when the negative electrolyte is oxidized partially in the initial stage.

4. In Figure 2, the author discusses the effects of the oxidation ratio of V2+ with air on the discharge capacity of VFBs, indicating a strong relativity with the net crossover ratio of V2+. And the exceeded oxidation may cause the accumulation of VO2+ and the capacity decay. However, the net flux of V2+ is dynamic based on the initial oxidation ratio of V2+ in practice. Please explain how to confirm the net flux of V2+ and optimize the oxidation ratio of the V2+.

5. On page 10, line 10, we find the wrong expression “V3+”. Please modify it.

6. In Figure 4d and 4e， it seems that the discharge capacity and voltage efficiency of the VFB coupled with V3.68+ electrolyte somehow exhibit regular fluctuations. Please explain it.

Reviewer: 2

Comments to the Author

"An Electrolyte with Elevated Average Valence for Suppressing the Capacity Decay of Vanadium Redox Flow Batteries" – review

An effect of an elevated average valence for suppressing the capacity decay of vanadium redox flow batteries was tested. Another method tested for the capacity suppression decay was the excessive V2+ ions air oxidation at the anolyte site. Electrolyte composition was investigated via UV-VIS spectrophotometry and the battery performance with different electrolytes was tested. The approach proposed is very original and even counter-intuitive, so the experimental data section should be revised in order to ensure the correctness of every statement of the research, even though it is supplementary. I think, the article can be accepted for publication is ACS general science after a major revision.

Comments to the experimental section of the supporting information:

1) From the supplementary materials it is not clear, what electrolyte was used for the V 3.68+ tests (Fig 4. (d-g) of the main article): was it the same initial electrolyte for both anolyte and catholyte or was it V 3.68+/V 3.5+ pair?

2) Tab S1: the table shown originally [3] was named as for the Nafion 115 membrane, not for 'Nafion serials membrane', and the diffusion coefficient was normalized to the cm2, not cm1 with the same quantitative values – this should be corrected or additional comments should be added. Even though those parameters were measured for Nafion 115 membrane, they were normalised to the membrane`s surface area, not the thickness, and direct application of these parameters to a twice thicker Nafion NR212 membrane is questionable.

3) Additional information to the calculations below SI1 should be added. What are the calculation metrics? I would assume, those are [ml * mole/liter(M)], which would not be correct. On the Fig. S1 the volumes are clearly 35 and 38 ml, what are the 38.15 and 41.85 numbers below? Is there any connection between these values?

'Initial content: 40*1.71+40*1.70=136.4

After 400 cycles :38.15*1.06+41.85*2.22=133.346'

- The calculation after Fig.S7 is named as (1), while the calculation after Fig.S1 is not – why?

- Write down in the description for the Fig.S1, S7 where is anolyte and where is catholyte. Only a very attentive reader could understand where is what from the Fig.S7 using those 'P'/'N' letters on the glass.

4) I would suggest changing the term 'cut-off voltage range' to the 'operating voltage range' in 1.3, 1st paragraph.

5) Please, name all the sections and the description correctly.

- '1.2 Characteristics' to 'UV-vis spectroscopy/spectrophotometry'.

- Make a section for the electrolyte preparation.

- Change the metrics of GF pieces to cm if you further normalize the pump speed to it in cm2.

- Add a section to the description of the remixing procedures, especially include the value for the ‘low discharge density’. Speaking of the discharge procedure here, why did not you use the constant voltage technique until the current stabilizes? At this point, you are having only crossover recharge and can tell the SOC is 0.

- Add a section for the viscosity measurements since you provide the experimental data.

- Add a description of Ox-C, Ox-4, Ox-0 meanings to pictures.

Fig.S2, Fig.S4 – name the graphs with the same line names, add the name meanings in the description.

6) What is meant in 2.2 ‘At the initial state, both the vanadium ions contents and the valence of the electrolytes are symmetry for the positive and negative sides. Hence, all the concentrations of vanadium ions on the two sides are the same at 50 SOC.’? What is the chemical state described here as 50 SOC? This thesis is confusing because ‘50 SOC’ state is, normally, an ideal case of half-charged battery having xV2+/xV3+ and 0V5+/ 0V4+ (zero) at the anolyte side vs xV5+/xV4+ and 0V2+/0V3+ (zero) at the catholyte side meaning different species concentrations on the two sides but equal concentration of each existing vanadium ion to each other; also this case excludes the preferential water transfer process.

7) Some additional information shall be added to S2, S4 figures because it is hard to tell the difference between them from the description. What is the role of the viscosity measurements for the research and why are the trends are different?

8) A table with all the settings and experimental data obtained shall be added.

9) Tab. S2: in order to make the available capacity calculations correct, you will need to change the metrics as (A h / L) as long as the system volume is not introduced and to add the formula explaining the values taken for the broad auditory of the journal that might not know the numbers used.

Also, consider using ‘mol L-1’ or ‘M’ through the article.

10) A part explaining the V2+ oxidizing process from the experimental setup perspective shall be added, especially in the case of Ox-C experiment. As for me, it raises a question of why did not all the V2+ oxidize? I would expect an accelerated capacity decay to a near-zero values in the case of a continuous air flow through the anolyte during the cycling.

11) The current density of 200 mA/cm2 is very high and reduces crossover significantly, while the research is exactly about this process. I would suggest such an experiment with 60 mA/cm current density, because it is hard to tell how close was the cycling to a symmetrical one from the SOC perspective, meaning asymmetric cycling, which is also a way of crossover control. In other words, the research might be considered as an addition to the asymmetric cycling procedure which makes the article very specific and would not reflect the general idea.

12) Make a general revision. I could have missed something as there are a lot of issues with the clearance.

Comments to the main article:

1) Fig.3: an information about the experiment shall be clarified: was it an experiment with initial V 3.5+? I would also suggest adding Ox-0, Ox-4, Ox-C meanings to the figure description.

2) Page 10, line 17: term ‘cut-off voltage’ describing the operating voltage might be confusing and I would suggest to change the term.

3) Fig.1(e): there is only V2+ crossover effect is demonstrated while vanadium ions of each valence have a significant effect on the process of V2+ surplus accumulation.

4) The theoretical explanation of the crossover effect on V2+ accumulation was accounted only at the 100% SOC and included only V2+ ions crossover, while there are four of them in total. Practically, the cycling was performed with only ~60-73% of the theoretical capacity depending on the answer to the 1st question of the previous comments section. This means that 0 or 100% SOC have never even been achieved during the cycling experiment, in fact, it was in the range of ~15%/20% – 75%/80% SOC meaning constant presence of V4+ ions having the second high diffusion coefficient after V2+ through the Nafion membrane [3] – this mechanism is also strongly affecting the process of V2+ accumulation. A more precise theoretical explanation of V 2+ accumulation is needed.

5) Fig.4 (d-e): An oscillation is observed in the case of V 3.68+. An explanation shall be added for the process as it looks like if it was air-oxidized or shunt-connected, which would make the comparison incorrect.

6) Since there are 2 methods proposed to reduce the effect of V2+ accumulation, I would suggest to change the accent of the article to highlight the air oxidation method as well.

(3) Sun C, Chen J, Zhang H, et al. Investigations on transfer of water and vanadium ions across Nafion membrane in an operating vanadium redox flow battery[J]. Journal of Power Sources, 2010, 195(3): 890-897. https://doi.org/10.1016/j.jpowsour.2009.08.041

Author's Response to Peer Review Comments:

Dear Editors and Reviewers,

Thank you for your and reviewers’ comments concerning our manuscript entitled “An Electrolyte with Elevated Average Valence for Suppressing the Capacity Decay of Vanadium Redox Flow Batteries” (oc-2022-01112j), which are very valuable and helpful for improving this paper. We considered these comments seriously and revised the manuscript based on them. The point-to-point responses to the comments are shown in the attachment.

Dear Editors and Reviewers,

Thank you for your and reviewers' comments concerning our manuscript entitled "An Electrolyte with Elevated Average Valence for Suppressing the Capacity Decay of Vanadium Redox Flow Batteries" (oc-2022-01112j), which are very valuable and helpful for improving this paper. We considered these comments seriously and revised the manuscript based on them. The point-to-point responses to the comments are listed as follows:

In this response document, text in *italic* style is the comments from the reviewers; text in regular style is our responses to the comments; revisions are highlighted in red color in both this letter, the revised manuscript, and the revised SI document; the literature and figures mentioned in the responses are sorted by order of appearance in this letter, while literature and figures used in the revised manuscript are sorted in the order of the manuscript.

Reviewer(s)' Comments to Author:

Reviewer: 1

Recommendation: Publish in ACS Central Science after minor revisions noted.

Comments:

This manuscript investigated the capacity decay behavior of VFBs caused by the crossover of the V^{2+}*. In addition to the vanadium ion loss of the negative electrolytes, authors propose the unavailable* V^{2+} *arisen from the double consumption of* VO_2^+ *induces the capacity decay in the long-term cycling. Herein, the manuscript demonstrates a simple strategy to suppress the capacity*

fading by enhancing the average valence of the original electrolyte ($V^{3.68+}$), indicating an improvement of 52.33% in the accumulated discharge capacity during 400 cycles of the VFB compared with the commercial electrolyte. The topic is interesting and the paper is well organized. Therefore, I suggest publishing this paper after considering the following comments:

1. In the abstract and introduction, research on membranes was mentioned and reviewed. However, this work is about electrolyte modification instead of the design of membranes. Please quote related references and review the progress in this field.

Response: Thanks for your valuable suggestions. The research on electrolytes of VRFBs is mainly focused on improving the energy efficiency by optimizing the supporting electrolytes,[17] such as mixing acid[18,19] and optimizing sulfuric acid concentration,[20] or increasing the high-temperature stability of $VO_2^+$and longer-period stability of V^{3+} under the high concentration with kind of additives. [21,22,23,24] The electrolyte study about improving the capacity retention of VRFBs is rarely reported. Additionally, we have cited the related references and briefly reviewed the studies on the electrolytes of VRFBs in the revised manuscript. Details as below:

[In the revised manuscript, Page4, line3]

Even though the electrolyte utilization of VRFBs can be enhanced to some extent by optimizing the sulfuric acid concentration,[18] adopting the mixing acid system,[19,20] and increasing the operating temperature (with some organic/inorganic additives), [21,22,23,24] such research is mainly focused on improving the energy efficiency of VRFBs. There is no research on improving the capacity retention of VRFBs by optimizing the configuration of electrolytes.

(17) Wu X, Liu J, Xiang X, Zhang J, Hu J, and Wu Y. Electrolytes for vanadium redox flow batteries[J]. *Pure Appl Chem*, 2014, 86(5): 661-669. DOI: 10.1515/pac-2013-1213

(18) Li L, Kim S, Wang W, Vijayakumar M, Nie Z, Chen B, Zhang J, Xia G, Graff G, Liu J and Yang Z. A stable vanadium redox-flow battery with high energy density for large-scale energy storage[J]. *Adv Energy Mater*, 2011, 1(3): 394-400. DOI: 10.1002/aenm.201100008

(19) Peng S, Wang N F, Wu X J, Liu S Q, Fang D, Liu Y N, and Huang K L. Vanadium species in CH3SO3H and H2SO4 mixed acid as the supporting electrolyte for vanadium redox flow battery[J]. *Int. J. Electrochem. Sci*, 2012, 7: 643-649. http://electrochemsci.org/papers/vol7/7010643.pdf

(20) Skyllas-Kazacos M, Cao L, Kazacos M, Kausar N, and Mousa A. Vanadium electrolyte studies for the vanadium redox battery—a review[J]. *ChemSusChem*, 2016, 9(13): 1521-1543. DOI:10.1002/cssc.201600102

(21) Vijayakumar M, Wang W, Nie Z, Sprenkle V, and Hu J. Elucidating the higher stability of vanadium (V) cations in mixed acid based redox flow battery electrolytes[J]. *J Power Sources*, 2013, 241: 173-177. DOI: 10.1016/j.jpowsour.2013.04.072

(22) Yang Y, Zhang Y, Liu T, and Huang J. Improved broad temperature adaptability and energy density of vanadium redox flow battery based on sulfate-chloride mixed acid by optimizing the concentration of electrolyte[J]. *J Power Sources*, 2019, 415: 62-68. DOI: 10.1016/j.jpowsour.2019.01.049

(23) Wang G, Chen J, Wang X, Tian J, Kang H, Zhu X, Zhang Y, Liu X, and Wang R. Influence of several additives on stability and electrochemical behavior of V (V) electrolyte for vanadium redox flow battery[J]. *J. Electroanal. Chem*, 2013, 709: 31-38. DOI: 10.1016/j.jelechem.2013.09.022

(24) Cao L, Skyllas-Kazacos M, Menictas C, and Noack J. A review of electrolyte additives and impurities in vanadium redox flow batteries[J]. *J Energy Chem*, 2018, 27(5): 1269-1291. 8.04.007 DOI: 10.1016/j.jechem.2018.04.007

2. In Figure 1c, based on the calculation, the concentration of the accumulated V^{2+} (0.05 M) seems too low to have a severe impact on the capacity retention according to the author's point of view, that the accumulated V^{2+} greatly decreases the discharge capacity of VRFBs after long-term cycling. Please explain.

Response: Thanks for your valuable comments.

Firstly, oxidizing the accumulated V^{2+} in the anolyte to V^{3+} with air can increase the active species, which can be utilized in the following charge/discharge cycles, and improve the voltage efficiency of VRFBs (Fig. 1(b)). The increased active species and voltage efficiency both contribute to the improvement of discharge capacity (Fig. 3(a)).

Additionally, in this work, the maximum discharge capacity of the VRFB under 200 mA cm^{-2} is 1.822 Ah, which accounts for 64.76% of the theoretical capacity of the electrolytes (40 mL, 1.7 mol L^{-1} $V^{3.5+}$), and the proportion decreases with capacity decay. Therefore, the changing of active species reflected in the variation of anolyte concentration is much smaller than that reflected in the variation of discharge capacity.

Furthermore, the concentration difference of V^{3+} displayed in Fig. 1(c) is smaller than its actual value of the accumulated V^{2+}. Because V^{2+} is very easy to be oxidized by air, which results in part of V^{2+} being oxidized to V^{3+} during the UV tests. In fact, the actual concentration of accumulated

V^{2+} should be measured without oxygen. However, it is inevitable to interfere with the air during the UV sample preparation and measurement under our laboratory's experimental conditions.

Besides, eliminating the accumulation of V^{2+} can reduce the crossover of V^{2+} from the negative to the positive sides and leads to the dynamic balance[1] between diffusion and convection arriving earlier, as shown in Figs. 4(f-g). Compared with the VRFB coupled with conventional electrolytes ($V^{3.50+}$), the concentration difference of the catholyte and anolyte of the VRFB coupled with the electrolytes of $V^{3.68+}$ decreases 0.18 M after 400 cycles.

Notably, the concentration differences of V^{3+} and VO^{2+} in Figs. 1(c-d) is only used to demonstrate that the unavailable V^{2+} is accumulated on the negative side during cycling. Hence, it does not affect the results and discussion in the following sections.

(1) Li L, Kim S, Wang W, Vijayakumar M, Nie Z, Chen B, Zhang J, Xia G, Graff G, et al. A stable vanadium redox-flow battery with high energy density for large-scale energy storage[J]. *Adv Energy Mater*, 2011, 1(3): 394-400. DOI: 10.1002/aenm.201100008

3. In Figure 1e, the schematic diagram vividly shows the formation of the unavailable V^{2+} and the capacity improvement through oxidation V^{2+} with air. However, no schematic diagram to illustrate the concept of the electrolyte with elevated average valence. If it is possible to add the schematic diagram of the evolution of vanadium ions during the charge/discharge processes when the negative electrolyte is oxidized partially in the initial stage.

Response: Thanks for your good advice. The schematic diagram of vanadium ions evolution of VRFBs with equilibrium ($V^{3.5+}$) and elevated valence electrolytes in the charge/discharge process are compared in Fig. S8. We also added the schematic diagram of vanadium ions evolution with

the elevated valence of VRFBs in the revised manuscript, as shown in Fig. 4(h). The VRFB with the elevated valence electrolyte generates the surplus VO_2^+ after a charge/discharge cycle (Fig. 4(h)). The surplus VO_2^+ eliminates the accumulation of V^{2+}, reduces the crossover of V^{2+} from the negative to the positive sides, and enables VRFBs to present a high capacity retention rate in cycling.

[In the revised manuscript, Page 12, line 1]

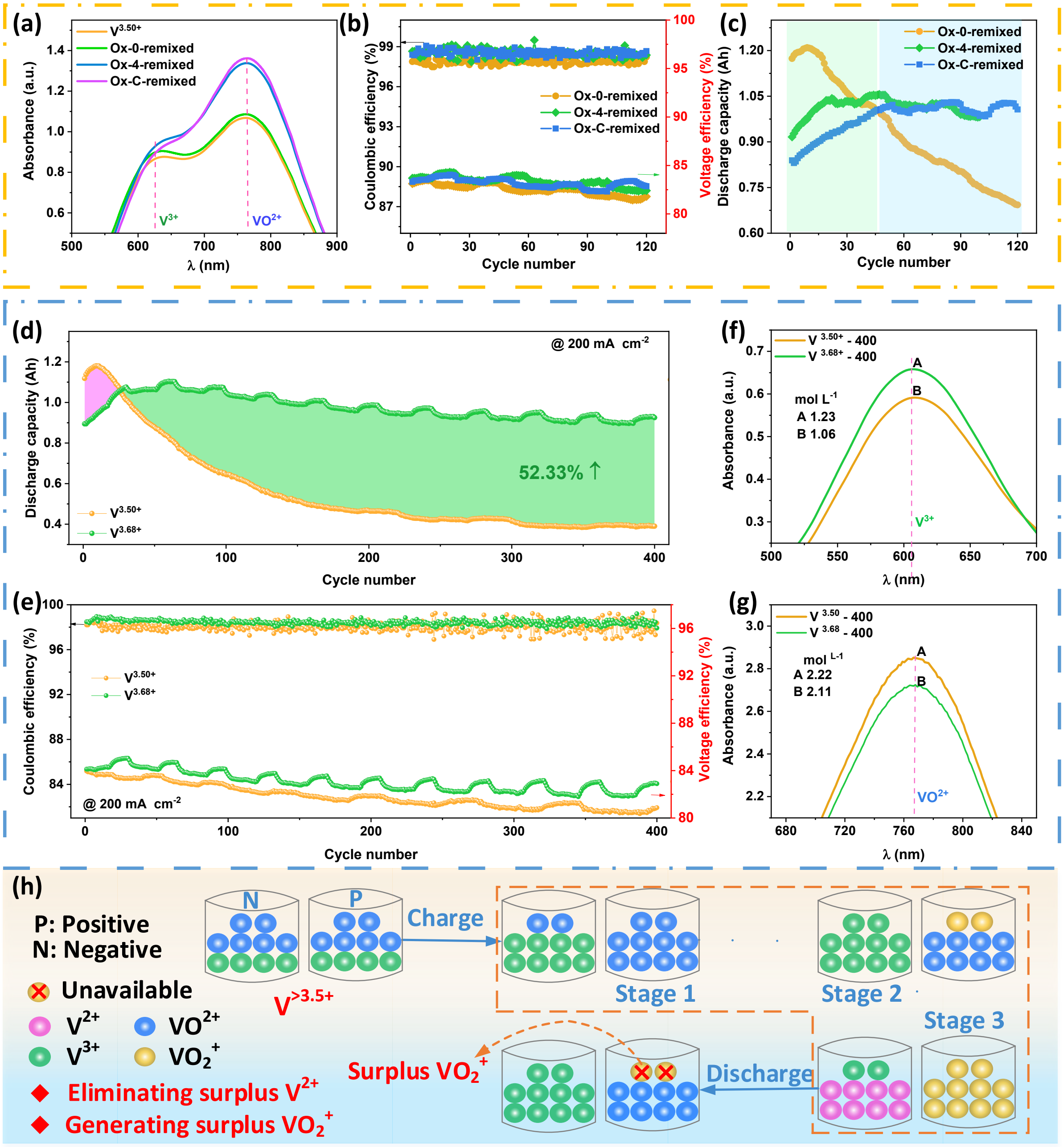

Fig. 4. (a) UV spectrums of the different electrolytes. (b) Coulombic efficiency, voltage efficiency, and (c) discharge capacity of VRFBs with remixed electrolyte for Ox-0, Ox-4, and Ox-C after 400 cycles. (d) Coulombic and voltage efficiency, and (d) discharge capacity of VRFBs with $V^{3.50+}$ and $V^{3.68+}$ electrolytes. (f-g) UV spectrums of the $V^{3.68+}$ and $V^{3.50+}$ electrolytes after 400 cycles. (h) The schematic diagram of vanadium ions evolution of the VRFB with elevated valence electrolytes ($V^{>3.5+}$) in the charge/discharge process.

Moreover, to make the vanadium ions evolution in the VRFBs with elevated valence electrolytes more straightforward in Fig. S8, we changed the title and added notations in the revised supporting information document. Details are shown below:

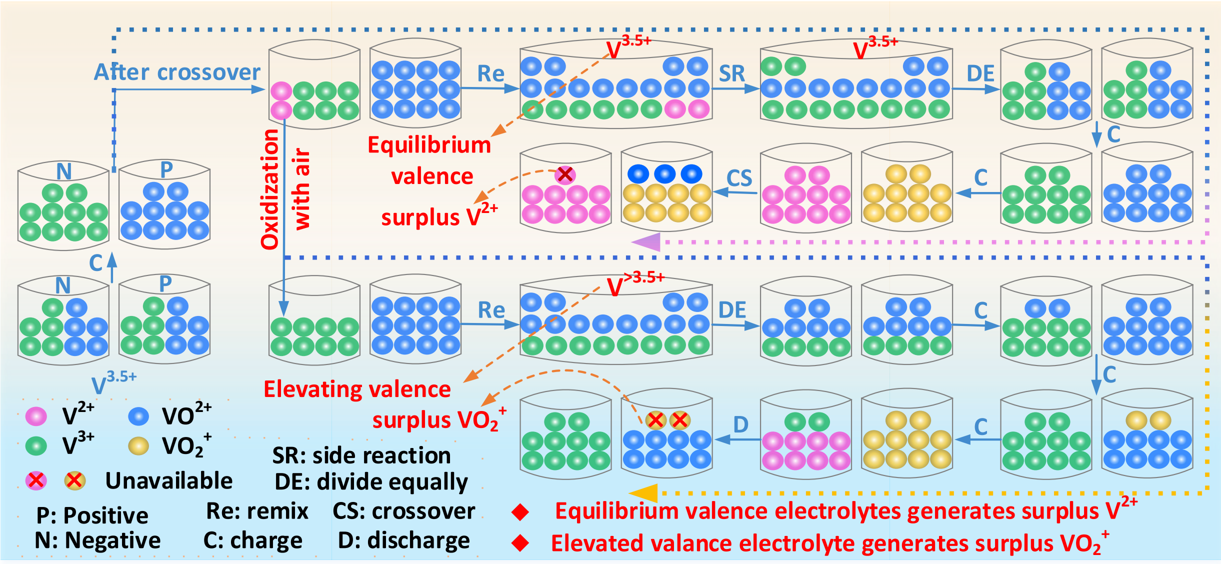

Fig. S8 The schematic of the vanadium ions evolution in the charge/discharge process of VRFBs with conventional remixed electrolytes and the oxidized electrolytes.

4. In Figure 2, the author discusses the effects of the oxidation ratio of V^{2+} with air on the discharge capacity of VFBs, indicating a strong relativity with the net crossover ratio of V^{2+}. And the exceeded oxidation may cause the accumulation of VO_2^+ and the capacity decay. However, the net flux of V^{2+} is dynamic based on the initial oxidation ratio of V^{2+} in practice. Please explain how to confirm the net flux of V^{2+} and optimize the oxidation ratio of the V^{2+}.

Response: Thanks for your valuable comments. The net flux of V^{2+} changes with the charge/discharge process, which affects the active species ratio (V^{2+}, V^{3+}, VO_2^+, and VO_2^+) and the maximum available capacity of VRFBs immediately. Let's assume that the anolyte of VRFB has 1 mol V^{2+}, the corresponding catholyte has 1 mol VO_2^+ at the initial stage, and the maximum discharge capacity is $1 \times F$ (F is Faraday constant F=96485 C mol^{-1}). Next, we define x as the net

flux of V^{2+} from the anolyte to the catholyte after cycling for a certain time. In that case, the content of V^{2+} in the anolyte for the fully charged VRFB is (1-x) mol, while the VO_2^+ and VO^{2+} contents in the catholyte are (1-2x) and 3x mol (the side reaction as Eq. (R1)). Hence, the maximum discharge capacity of the VRFB after x mol V^{2+} net flux from the anolyte to the catholyte is (1-2x)×F. Moreover, 1 mol V^{2+} consumes 1 mol VO_2^+ in the discharge process. Therefore, it remains x mol V^{2+} in the anolyte after fully discharging the VRFB, which is equal to the net flux of V^{2+} crossover from the anolyte to the catholyte.

Therefore, in the VRFB, the net flux of V^{2+} after running for a certain time is equal to the residual V^{2+} in the anolyte after being fully discharged (discharge the VRFB with gradually decreased current densities). The optimal oxidized ratio of V^{2+} is oxidizing all the accumulated unavailable V^{2+} to V^{3+} with air without sacrificing the available V^{2+}. Thus, the optimal oxidized amount of V^{2+} is equal to the residual amount of V^{2+} in the anolyte after being fully discharged. That means we only need to fully discharge the VRFB and completely oxidize the anolyte with air without additional calculation in practice.

$$V^{2+} + 2VO_2^+ + 2H^+ \rightarrow 3VO^{2+} + H_2O \qquad \text{(R1)}$$

5. On page 10, line 10, we find the wrong expression "V^{3+}". Please modify it.

Response: Thank you very much. We are very sorry for the typo. We have corrected it in the revised manuscript.

6. In Figure 4d and 4e, it seems that the discharge capacity and voltage efficiency of the VFB coupled with $V^{3.68+}$ electrolyte somehow exhibit regular fluctuations. Please explain it.

Response: Thanks for your valuable comments. The regular fluctuation of the discharge capacity and voltage efficiency of the VRFB coupled with $V^{3.68+}$ is caused by the regular variation in room

temperature. In our laboratory, the temperature changes regularly with time, which influences the battery efficiencies and discharge capacity regularly. Additionally, the changing temperature effects are also reflected in the tests of VRFB coupled with $V^{3.50+}$ and present a similar fluctuation amplitude, as shown below. However, the fluctuation frequency with the cycle number of the VRFB coupled with $V^{3.50+}$ is much smaller than that coupled with $V^{3.68+}$ because the former displays a much smaller discharge capacity than the latter (Fig. 4(d)). Therefore, the VRFB coupled with $V^{3.50+}$ runs more cycles at the same term than that coupled with $V^{3.68+}$. Thus, the VRFB coupled with $V^{3.68+}$ presents more signific fluctuation with cycling than with $V^{3.50+}$.

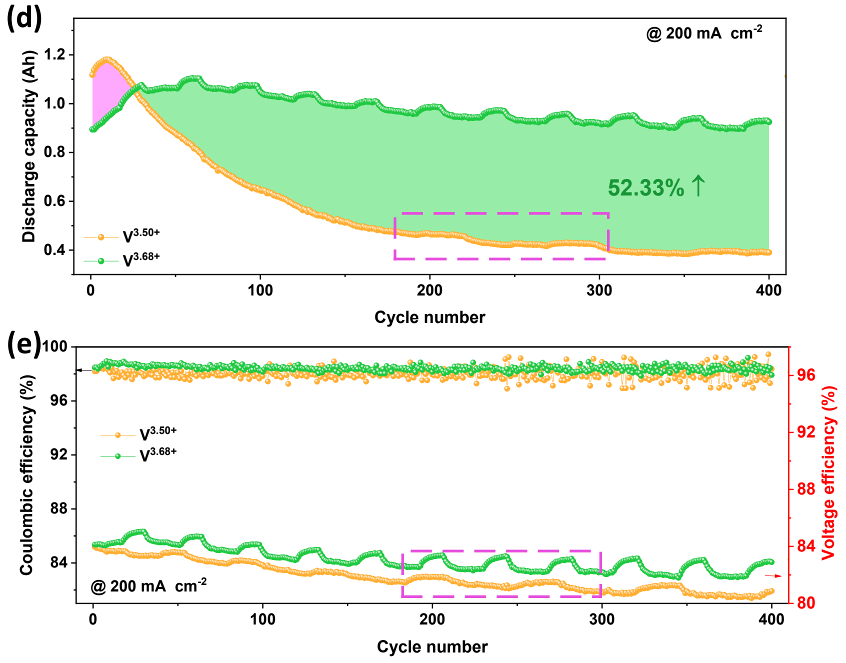

Fig. 4. (d) The discharge capacity and (e) coulombic/voltage efficiency of VRFBs with $V^{3.50+}$ and $V^{3.68+}$.

We thank the reviewer for his/her important comments. We have carefully revised the manuscript according to these valuable suggestions.

Reviewer: 2

Recommendation: Reconsider after major revisions noted.

An effect of an elevated average valence for suppressing the capacity decay of vanadium redox flow batteries was tested. Another method tested for the capacity suppression decay was the excessive V^{2+} ions air oxidation at the anolyte side. Electrolyte composition was investigated via UV-VIS spectrophotometry and the battery performance with different electrolytes was tested. The approach proposed is very original and even counter-intuitive, so the experimental data section should be revised in order to ensure the correctness of every statement of the research, even though it is supplementary. I think, the article can be accepted for publication is ACS general science after a major revision.

Comments to the main article:

1. Fig.3: an information about the experiment shall be clarified: was it an experiment with initial $V^{3.5+}$? I would also suggest adding Ox-0, Ox-4, Ox-C meanings to the figure description.

Response: Thanks for your valuable comments. Yes, the experiments in Fig. 3 were conducted with the commercial electrolytes ($V^{3.5+}$), and we also added the relevant information in the revised manuscript, as shown below.

In Fig. 3, the Ox-0 presents the VRFB operated with nitrogen protection without V^{2+} oxidized by air during 400 cycles. Ox-4 means the VRFB with 4 times air oxidization of V^{2+} at the 50th, 100th, 200th, and 300th cycles during cycling. In Figs. 3(c-d), Ox-C represents the VRFB in which the negative electrolyte is continually oxidized with air during cycling. We did not place the meanings of Ox-0, Ox-4, and Ox-C in the title of Fig. 3 to avoid redundancy. Instead, we placed the

explanation of Ox-0, Ox-4, and Ox-C below Fig. 3 in the manuscript. Besides, we also added the meanings of Ox-0, Ox-4, and Ox-C in the revised supporting information (Section 1.6, Page S3, line 10).

[In the revised manuscript, Page 9, line 9]

To further examine the specific effect of oxidizing V^{2+} with air on the capacity of VRFB, the VRFBs with the commercial electrolyte ($V^{3.50+}$) are tested with and without air oxidization during cycling, as shown in Fig. 3.

2. Page 10, line 17: term 'cut-off voltage' describing the operating voltage might be confusing and I would suggest to change the term.

Response: Thanks for your suggestion. We have changed the 'cut-off voltage range' to the 'operating voltage range' in the revised manuscript and supporting information (Section 1.6, Page S3, line 12).

3. Fig.1(e): there is only V^{2+} crossover effect is demonstrated while vanadium ions of each valence have a significant effect on the process of V^{2+} surplus accumulation.

Response: Thanks for your valuable comments. The side reactions (Eqs. S1-S6) will occur when vanadium ions cross the membrane from one side to the other side, and the effect of crossover on vanadium ions' evolution in the charge/discharge process are depicted in Fig. S3. Fig. S3(a) shows that although the crossover of V^{2+} and V^{3+} consumes different amounts of VO_2^+ in the catholyte, both result in the same amount of unavailable V^{2+} accumulation in the anolyte finally. Similarly, the crossover of VO^{2+} and VO_2^+ consumes different amounts of V^{2+} in the anolyte and both result in the same amount of unavailable VO_2^+ accumulation in the catholyte, as shown in Fig. S3(b).

The results mean the accumulated amount of unavailable V^{2+} is a constant at a certain net flux of electrolyte, no matter how much the vanadium ions (V^{2+}, V^{3+}, VO^{2+}, VO_2^+) contribute to the crossover separately. Moreover, the concentration and volume of the electrolyte increase on the positive side and decrease on the negative side with cycling[1] due to the much higher diffusion rate across the Nafion series membranes of V^{2+} than that of other vanadium ions.[2] The electrolytes' changing during VRFB cycling results in the surplus of V^{2+} on the negative side.[3,4]

Therefore, in this work, we used the net flux of V^{2+} (the amount of vanadium ions (V^{2+}/V^{3+}) crossover from the anolyte to the catholyte, subtract the amount of vanadium ions (VO^{2+}/VO_2^+) crossover from the catholyte to the anolyte) to depict the effects of vanadium ions crossover on the accumulation process of unavailable V^{2+} for brevity. We also modified the related contents in the revised manuscript to make it more straightforward.

Side reactions for the vanadium ions transport from the anolyte to the catholyte:

$$V^{2+} + 2VO_2^+ + 2H^+ \rightarrow 3VO^{2+} + H_2O \tag{S1}$$

$$V^{2+} + VO^{2+} + 2H^+ \rightarrow 2V^{3+} + H_2O \tag{S2}$$

$$V^{3+} + VO_2^+ \rightarrow 2VO^{2+} \tag{S3}$$

Side reactions for the vanadium ions transport from the catholyte to the anolyte:

$$VO_2^+ + 2V^{2+} + 2H^+ \rightarrow 3VO^{2+} + H_2O \tag{S4}$$

$$VO_2^+ + V^{3+} \rightarrow 2VO^{2+} \tag{S5}$$

$$VO^{2+} + V^{2+} + 2H^+ \rightarrow 2V^{3+} + H_2O \tag{S6}$$

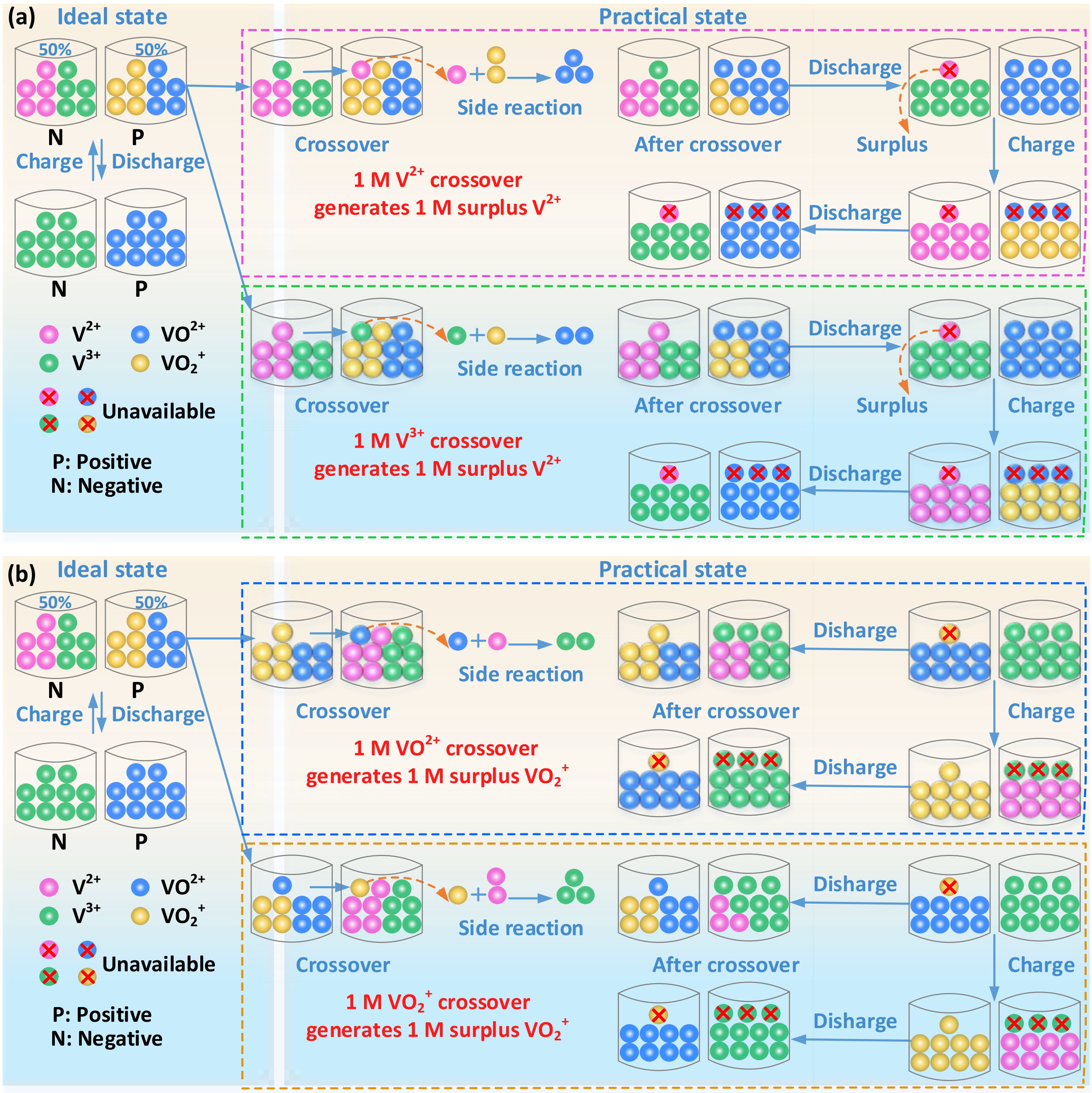

Fig. S3 The schematic of (a) V^{2+}/V^{3+} and (b) VO^{2+} /VO_2^+ crossover effect on the ions' evolution in the charge/discharge process.

[In the revised manuscript, page 7, line20]

First, we analyzed all the vanadium ions crossover effects on the ions' evolution in the charge/discharge process separately, as shown in Fig. S3. Fig. S3(a) shows that although the crossover of V^{2+} and V^{3+} consumes different amounts of VO_2^+on the positive side, both result in

the same amount of unavailable V^{2+} accumulation on the negative side finally. Similarly, the crossover of VO^{2+} and VO_2^+ consuming a different amount of V^{2+} on the negative side, both result in the same amount of unavailable VO_2^+ accumulation on the positive side finally, as shown in Fig. S3(b). That means the accumulated amount of unavailable V^{2+} is a constant at a certain net flux of electrolyte, no matter how much the vanadium ions (V^{2+}, V^{3+}, VO^{2+}, VO_2^+) contribute to the crossover separately. Moreover, the concentration and volume of the electrolyte increase on the positive side and decrease on the negative side with cycling[7] due to the much higher diffusion rate across the Nafion series membranes of V^{2+} than other vanadium ions.[3] The electrolytes' changing during VRFB cycling results in the surplus of V^{2+} on the negative side.[4,5] Therefore, we used the net flux of V^{2+} (the amount of vanadium ions (V^{2+}/ V^{3+}) crossover from the negative to the positive sides subtract the amount of vanadium ions (VO^{2+}/VO_2^+) crossover from the positive to the negative sides) to depict the effects of vanadium ions crossover on the ions' evolution in the charge/discharge process.

(3) Vrána J, Charvát J, Mazúr P, Belsky P, Dundalek J, Pocedic J, and Kosek J. Commercial perfluorosulfonic acid membranes for vanadium redox flow battery: Effect of ion-exchange capacity and membrane internal structure[J]. *J Membrane Sci*, 2018, 552: 202-212. DOI: 10.1016/j.jpowsour.2019.227503

(4) Song Y, Li X, Xiong J, Yang L, Pan G, Yan C, and Tang A. Electrolyte transfer mechanism and optimization strategy for vanadium flow batteries adopting a Nafion membrane[J]. *J Power Sources*, 2020, 449: 227503. DOI: 10.1016/j.jpowsour.2019.227503

(5) Tang A, Bao J, Skyllas-Kazacos M. Dynamic modelling of the effects of ion diffusion and side reactions on the capacity loss for vanadium redox flow battery. *J Power Sources*. 2011;196(24):10737–47. DOI: 10.1016/j.jpowsour.2016.02.018

(7) Luo Q, Li L, Wang W, Nie Z, Wei X, Li B, Chen B, Yang Z, and Sprenkle V. Capacity decay and remediation of nafion-based all-vanadium redox flow batteries. *ChemSusChem*. 2013;6(2):268–74. DOI: 10.1002/cssc.201200730

4. The theoretical explanation of the crossover effect on V^{2+} accumulation was accounted only at the 100% SOC and included only V^{2+} ions crossover, while there are four of them in total. Practically, the cycling was performed with only ~60-73% of the theoretical capacity depending on the answer to the 1st question of the previous comments section. This means that 0 or 100% SOC have never even been achieved during the cycling experiment, in fact, it was in the range of ~15%/20% – 75%/80% SOC meaning constant presence of V^{4+} ions having the second high diffusion coefficient after V^{2+} through the Nafion membrane [3] – this mechanism is also strongly affecting the process of V^{2+} accumulation. A more precise theoretical explanation of V^{2+} accumulation is needed.

Response: Thanks for your valuable comments. As the answer to question 3, although four kinds of vanadium ions' crossover can influence the unavailable V^{2+} accumulation in the anolyte, the crossover of V^{2+} and V^{3+} generates the same amount of unavailable V^{2+} in the anolyte finally, and the crossover of VO^{2+} and VO_2^+ generate the same amount of unavailable VO_2^+ in the catholyte. The results mean the accumulated amount of unavailable V^{2+} is a constant with a certain net flux of electrolyte, no matter how much the vanadium ions (V^{2+}, V^{3+}, VO^{2+}, VO_2^+) contribute to the crossover separately. Moreover, V^{2+} presents the highest diffusion rate across Nafion series membranes than other vanadium ions,[1] which results in the net flux of electrolyte transport from the anolyte to the catholyte[2] and the surplus of V^{2+} in the anolyte.[3,4] Therefore, in this work, we used the net flux of V^{2+} (the amount of vanadium ions (V^{2+}/V^{3+}) crossover from the anolyte to the catholyte subtract the amount of vanadium ions (VO^{2+}/VO_2^+) crossover from the anolyte to the

catholyte) to depict and theoretically calculate the effects of vanadium ions crossover on the accumulation process of unavailable V^{2+}.

In practice, the capacity of a VRFB with a certain amount of electrolyte is influenced by the electrolyte flow rate, current density, and other working conditions, which results in the capacity being lower than the electrolytes' theoretical capacity. In this work, the theoretical capacity of the electrolytes (40 mL, 1.7 mol L^{-1} $V^{3.5+}$) is 1.822 Ah, and the maximum discharge capacity of the VRFB in the experiment is 1.180 Ah (64.76% SOC).

As the reviewer comments, the SOC of VRFB affects the vanadium ions crossover[5] and influences the V^{2+} accumulation in the charge/discharge process. However, as mentioned above, the accumulated amount of unavailable V^{2+} is a constant at a certain net flux of the electrolyte, no matter how much the vanadium ions (V^{2+}, V^{3+}, VO^{2+}, VO_2^+) contribute to the crossover separately. In this work, we used the net flux of V^{2+} to present the net flux of all vanadium ions crossover from the anolyte to the catholyte. Then, we used the net flux of V^{2+} to depict and calculate the effects of vanadium ions crossover on the accumulation process of unavailable V^{2+}. Therefore, the SOC does not influence the calculated results of Fig. 2 in the manuscript. In this work, we used 100% SOC to depict the accumulation process of unavailable V^{2+} because it is easy to present the amount of unavailable V^{2+} during the ions' evolution process.

(1) Vrána J, Charvát J, Mazúr P, Belsky P, Dundalek J, Pocedic J, and Kosek J. Commercial perfluorosulfonic acid membranes for vanadium redox flow battery: Effect of ion-exchange capacity and membrane internal structure[J]. *J Membrane Sci*, 2018, 552: 202-212. DOI: 10.1016/j.jpowsour.2019.227503

(2) Luo Q, Li L, Wang W, Nie Z, Wei X, Li B, Chen B, Yang Z, and Sprenkle V. Capacity decay and remediation of nafion-based all-vanadium redox flow batteries. *ChemSusChem*. 2013;6(2):268–74. DOI: 10.1002/cssc.201200730

(3) Song Y, Li X, Xiong J, Yang L, Pan G, Yan C, and Tang A. Electrolyte transfer mechanism and optimization strategy for vanadium flow batteries adopting a Nafion membrane[J]. *J Power Sources*, 2020, 449: 227503. DOI: 10.1016/j.jpowsour.2019.227503

(4) Tang A, Bao J, Skyllas-Kazacos M. Dynamic modelling of the effects of ion diffusion and side reactions on the capacity loss for vanadium redox flow battery. *J Power Sources*. 2011;196(24):10737–47. DOI: 10.1016/j.jpowsour.2016.02.018

5. Fig.4 (d-e): An oscillation is observed in the case of $V^{3.68+}$. *An explanation shall be added for the process as it looks like if it was air-oxidized or shunt-connected, which would make the comparison incorrect.*

Response: Thanks for your valuable suggestion. The regular fluctuation of the discharge capacity and voltage efficiency of the VRFB with $V^{3.68+}$ electrolyte is caused by the regular variation in room temperature. In our laboratory, the temperature changes regularly with time, which influences the battery efficiencies and discharge capacity to some extent. Additionally, the changing temperature effects are also reflected in the tests of VRFB with $V^{3.50+}$ electrolyte and present a similar fluctuation amplitude, as shown below. However, the fluctuation frequency with the cycle number of the VRFB with $V^{3.50+}$ is much smaller than that with $V^{3.68+}$ because the former displays a much smaller discharge capacity than the latter (Fig. 4(d)). Therefore, the VRFB with $V^{3.50+}$ electrolyte runs more cycles under the same term than that with $V^{3.68+}$ electrolyte. Thus, the VRFB with $V^{3.68+}$ electrolyte presents more signific fluctuation with cycling than with $V^{3.50+}$ electrolyte. We have added an explanation of the oscillation phenomenon in the revised manuscript.

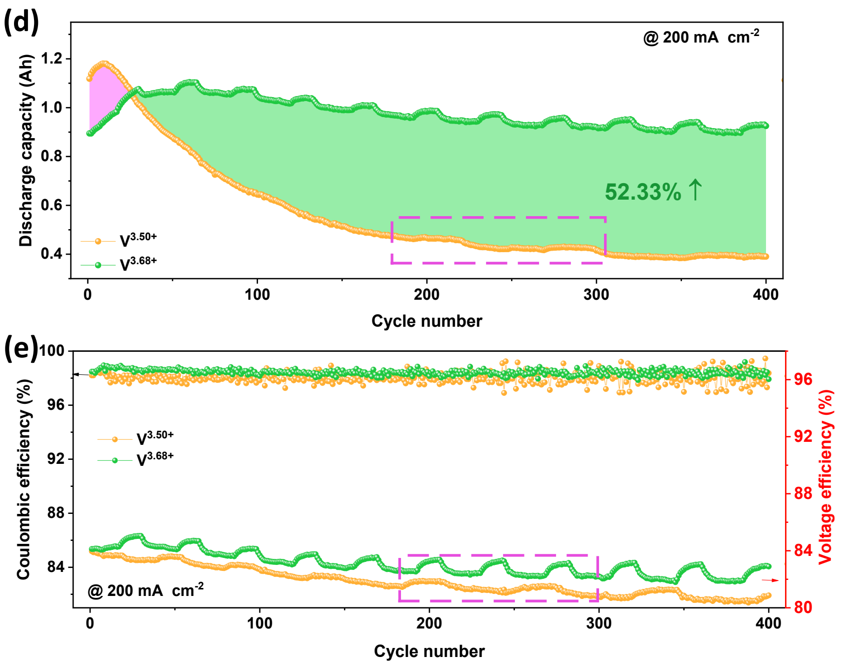

Fig. 4. (d) Discharge capacity and (e) coulombic/voltage efficiency of VRFBs with $V^{3.68+}$ and $V^{3.50+}$ electrolytes.

[In the revised manuscript, page 15, line20]

Besides, the discharge capacity and voltage efficiency of the VRFB coupled with $V^{3.68+}$ present a larger fluctuation frequency with cycling than that of VRFB coupled with $V^{3.50+}$, but they display a similar fluctuation amplitude. The reason for the fluctuation is the varying room temperature, which changes regularly with time in our laboratory. However, the discharge capacity of the VRFB coupled with $V^{3.50+}$ is much smaller than that with $V^{3.68+}$ after 200 cycles (Fig. 4(d)), leading to a much smaller fluctuation frequency with cycles for the former. Moreover, the rapid capacity decay of the VRFB with $V^{3.50+}$ in the first 150 cycles also concealed the fluctuation phenomenon.

6. Since there are 2 methods proposed to reduce the effect of V^{2+} accumulation, I would suggest to change the accent of the article to highlight the air oxidation method as well.

Response: Thanks for your important suggestion. According to your suggestion, we highlighted that oxidizing the unavailable V^{2+} to V^{3+} with air could significantly improve the discharge capacity of VRFBs in the conclusion section. However, the inside of the VRFB system is generally isolated from the external environment to ensure the sealing system and avoid additional side reactions in practical applications. Therefore, although both methods can effectively reduce the capacity decay rate of VRFBs, the utilization of electrolytes with an elevated average valance is a preferred method.

Moreover, the air oxidization method proposed in this work is mainly used to demonstrate the effects of the accumulated unavailable V^{2+} on the capacity of VRFBs and introduce the electrolytes with elevated average valence. In short, we think elevating the average valence of electrolytes is more meaningful and easier to handle for the engineering application of VRFBs.

Comments to the experimental section of the supporting information:

1. From the supplementary materials it is not clear, what electrolyte was used for the $V^{3.68+}$ *tests (Fig 4. (d-g) of the main article): was it the same initial electrolyte for both anolyte and catholyte or was it* $V^{3.68+}/V^{3.5+}$ *pair?*

Response: Thanks for your suggestion. In this work, the $V^{3.68+}$ electrolytes were used as the catholyte and anolyte of VRFBs in the battery tests (noted as $V^{3.68+}$). We have added the detail description of this section in the revised supporting information (Section 1.6).

2. Tab S1: the table shown originally [3] was named as for the Nafion 115 membrane, not for 'Nafion serials membrane', and the diffusion coefficient was normalized to the cm^2*, not* cm^1 *with the same quantitative values – this should be corrected or additional comments should be added. Even though those parameters were measured for Nafion 115 membrane, they were normalized to*

the membrane`s surface area, not the thickness, and direct application of these parameters to a twice thicker Nafion NR212 membrane is questionable.

Response: Thanks for your good comments. I am sorry for the typo on the diffusion coefficient. We have corrected the metric of diffusion coefficient, as well as the title of Tab. S1 in the revised supporting information. Additionally, the diffusion coefficient of vanadium ions is obtained in the reference of [3] as below:

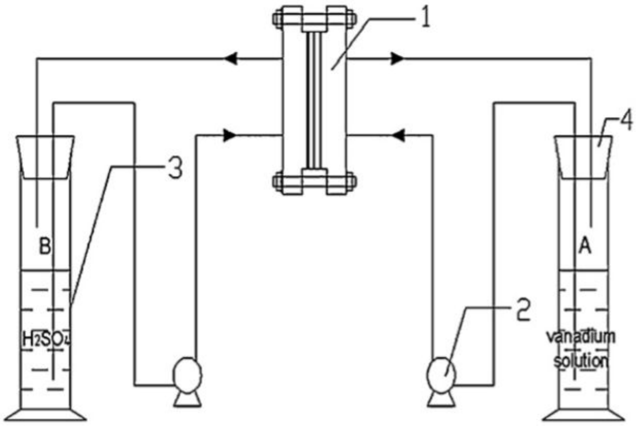

1 - dialysis cell; 2 - pump; 3 - measuring cylinder; 4 - rubber plug.

Fig. R1Schematic diagram of the dialysis cell employed for determining the diffusion coefficients of vanadium ions.[3]

$$D = \ln\left(\frac{C_A}{C_A - C_B}\right)\frac{V_B L}{At} \tag{R2}$$

Where D is the diffusion coefficient of vanadium ions ($m^2\ s^{-1}$); A is the effective area of the membrane (m^2); L is the thickness of the membrane (m); C_A is the concentration of vanadium ions in enrichment side (mol L^{-1}); C_B is the concentration of vanadium ions in deficiency side (mol L^{-1}); and t is the test time (s).

According to Eq. R2, the diffusion coefficient of vanadium ions can be calculated with the membrane thickness (L) in the reference.[3] Moreover, among the Nafion series membranes, Nafion 115 and Nafion 212 use the same raw material but have different thicknesses (Nafion 115: 127 µm, Nafion 212: 58 µm).[4] Therefore, it is reasonable to use the vanadium diffusion rate of Nafion 115 as Nafion 212. Besides, in this work, the vanadium diffusion rates of vanadium ions (V^{2+}, V^{3+}, VO^{2+}, VO_2^+) across Nafion 212 are only used to explain the reason for the net flux of electrolyte transport from the anolyte to the catholyte. The phenomenon of electrolytes transport from the anolyte to the catholyte after cycling has been validated in the VRFBs equipped with Nafion series membranes,[5-8] and the experiment in this work.

(3) Sun C, Chen J, Zhang H, Han X, and Luo Q. Investigations on transfer of water and vanadium ions across µNafion membrane in an operating vanadium redox flow battery[J]. *J Power Sources*, 2010, 195(3): 890-897. DOI: 10.1016/j.jpowsour.2009.08.041

(4) https://www.fuelcellstore.com/

(5) Jiang B, Wu L, Yu L, Qiu X, and Xi J. A comparative study of Nafion series membranes for vanadium redox flow batteries[J]. *J Membrane Sci*, 2016, 510: 18-26. DOI:10.1016/j.memsci.2016.03.007

(6) Wang K, Liu L, Xi J, et al. Reduction of capacity decay in vanadium flow batteries by an electrolyte-reflow method[J]. *J Power Sources*, 2017, 338: 17-25.

(7) Luo Q, Li L, Wang W, Xi J, Wu Z, and Qiu X. Capacity decay and remediation of nafion-based all-vanadium redox flow batteries[J]. *ChemSusChem*, 2013, 6(2): 268-274. DOI: 10.1002/cssc.201200730

(8) Park J H, Park J J, Park O O, and Yang J. Capacity decay mitigation by asymmetric positive/negative electrolyte volumes in vanadium redox flow batteries[J]. *ChemSusChem*, 2016, 9(22): 3181-3187. DOI: /10.1002/cssc.201601110

*3. Additional information to the calculations below SI1 should be added. What are the calculation metrics? I would assume, those are [ml * mole/liter(M)], which would not be correct. On the Fig. S1 the volumes are clearly 35 and 38 ml, what are the 38.15 and 41.85 numbers below? Is there any connection between these values?*

*'Initial content: 40*1.71+40*1.70=136.4*

*After 400 cycles :38.15*1.06+41.85*2.22=133.346'*

Response: Thanks for your question. The metric of the electrolytes volume is mL, and the metric of electrolytes concentration is M.

Initially, 40 mL 1.71 M V^{3+} and 40 mL 1.70 M VO^{2+} were used as the anolyte and catholyte for VRFB respectively. Thus, the total vanadium ions can be calculated below.

$$40 \times 1.71 + 40 \times 1.70 = 136.4 \text{ mM} \quad \text{(S8)}$$

Let's assume that the total volume of the anolyte and catholyte in VRFBs is the same before and after cycling. However, the electrolytes cannot wholly be pumped out from the VRFB after cycling due to the electrode absorption and the residual in pipes and channels, resulting in the total electrolytes after cycling obviously less than that at the initial stage, as shown in Fig. S4(c) (catholyte: 38.5 mL, anolyte: 34.8 mL). Next, suppose the electrolyte consumption (residual in the electrodes, pipes, and channels) is the same on the positive and negative sides. Thus, the electrolyte consumption in each half-cell can be calculated below.

$$\frac{1}{2}\big((40+40)-(33.8+38.5)\big)=3.35\ \text{mL} \tag{S9}$$

Therefore, the actual volume of electrolytes on the negative and positive side of the VRFB after cycling can be obtained as below:

$$\text{Positive side: } 3.35+38.5=41.85\ \text{mL} \tag{S10}$$

$$\text{Negativ side: } 3.35+33.8=37.15\ \text{mL} \tag{S11}$$

Hence, the total vanadium ion in the electrolyte of VRFB after 400 cycles can be calculated as Eq. (S12). And the calculated result is very close to that at the initial stage.

$$37.15\times 1.06+41.85\times 2.22=132.286\ \text{mM} \tag{S12}$$

We have also added these descriptions in the revised supporting information.

4. The calculation after Fig. S7 is named as (1), while the calculation after Fig. S1 is not – why?

Response: Thanks for your suggestion. We have numbered all the equations in the revised supporting information.

5. Write down in the description for the Fig. S1, S7 where is anolyte and where is catholyte. Only a very attentive reader could understand where is what from the Fig. S7 using those 'P'/'N' letters on the glass.

Response: Thanks for your suggestion. We have added the electrolyte information in the revised supporting information in Fig. S1 and Fig. S7.

6. I would suggest changing the term 'cut-off voltage range' to the 'operating voltage range' in 1.3, 1st paragraph.

Response: Thanks for your suggestion. We have changed the 'cut-off voltage range' to the 'operating voltage range' in the revised supporting information (Section 1.3, Page S2, line 12).

7. Please, name all the sections and the description correctly.

Response: Thanks for your suggestion. We have renamed the related sections in the revised supporting information.

8. – Replace '1.2 Characteristics' to 'UV-vis spectroscopy/spectrophotometry'.

Response: Thanks for your suggestion. We have replaced 'Characteristics' with UV-vis spectroscopy in the revised supporting information.

9. - Make a section for the electrolyte preparation.

Response: Thanks for your suggestion. We have added Section 1.3, which was named electrolyte preparation, and described the electrolyte preparation process more specifically in the revised supporting information. Details as below:

[In the revised supporting information, Section 1.3, Page S2, line 3]

1.3 Electrolyte preparation

The 1.70 M VO^{2+}/ 3.00 M H_2SO_4 electrolyte was obtained by charging the commercial electrolyte (1.70 M $V^{3.50+}$ /3.00 M H_2SO_4). The electrolyte with the valence of $V^{3.68+}$ was obtained by remixing 52.0 mL commercial electrolyte ($V^{3.50+}$) with 28.0 mL VO^{2+} electrolyte, and the actual valence state of the electrolyte is $V^{3.675+}$. In this work, we noted $V^{3.675+}$ as $V^{3.68+}$ to keep the form consistent with $V^{3.50+}$.

10. - Change the metrics of GF pieces to cm if you further normalize the pump speed to it in cm^2.

Response: Thanks for your suggestion. We have changed the metrics of GF to cm in the revised supporting information.

11. - Add a section to the description of the remixing procedures, especially include the value for the 'low discharge density'. Speaking of the discharge procedure here, why did not you use the constant voltage technique until the current stabilizes? At this point, you are having only crossover recharge and can tell the SOC is 0.

Response: Thanks for your suggestion. We have added a Section 1.4 named 'Remixing electrolytes' in the revised supporting information, details as below:

In this work, we used Arbin 179539 (Arbin instruments, USA) to record the experiment data, which has a maximum working current of 10 A. However, the discharge current exceeds 10 A at the initial stage when we use the constant voltage model to discharge the VRFB. Therefore, in this work, we adopted the gradient decreasing current density (312.5-200-125-62.5-50-18.75 mA cm^2) to discharge VRFBs until the discharge voltage arrives at 0 V. Moreover, in the constant voltage discharge model, the SOC value of electrolytes can be regarded as 0 when the discharge current is stable at a value (close to 0). In the constant current model, the SOC value of electrolytes can be regarded as 0 when the discharge voltage arrives at 0 at a small current density (18.75 mA cm^2 is very small in VRFBs).

[In the revised supporting information, Section 1.4, Page S2, line 7]

Before remixing the electrolytes on the positive and negative sides, a gradient decreasing current density (312.5-200-125-62.5-50-18.75 mA cm^2) is conducted to discharge the VRFBs (stopped at 0 V) to 0 SOC. Next, inverting the pump speed to pump electrolytes into tanks; third, connecting the catholyte outlet to the anolyte outlet and pumping all the catholyte to the anolyte, and remixing the electrolytes. Finally, shake well the remixed electrolytes and divide the electrolytes equally after waiting for 1 h. The remixing electrolytes process is depicted in Fig. S1.

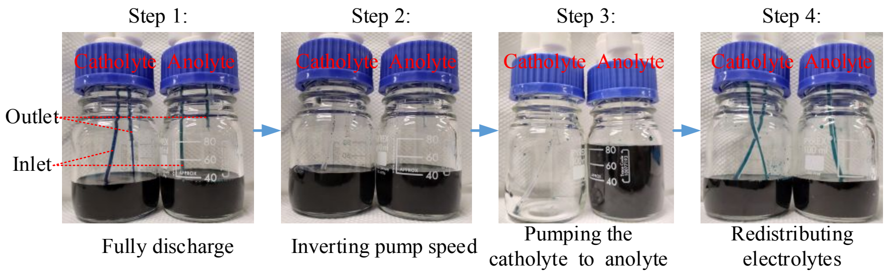

Fig. S1. Remixing electrolytes procedures.

12. - Add a section for the viscosity measurements since you provide the experimental data.

Response: Thanks for your suggestion. We have added a Section 1.5 named 'Viscosity measurement' in the revised supporting information, details as below:

[In the revised supporting information document, Section 1.5, Page S2, line 18]

The viscosity of vanadium electrolytes is tested with the 1834 Ubbelohde viscometer in a thermostat HS0100 (Lorderan, China).

13. - Add a description of Ox-C, Ox-4, Ox-0 meanings to pictures. Fig.S2, Fig.S4

Response: Thanks for your suggestion. The Ox-0 presents the VRFB operated with nitrogen protection without V^{2+} oxidized by air during 400 cycles. Ox-4 means the VRFB with 4 times air oxidization of V^{2+} at the 50th, 100th, 200th, and 300th cycles during cycling. Ox-C represents the VRFB in which the anolyte is oxidized continually with air during cycling. We have added the related description in the revised supporting information.

14. – name the graphs with the same line names, add the name meanings in the description.

Response: Thanks for your suggestion. We have renamed the legends in graphs and made them consistent. The description of the related legends is also added in the revised supporting information.

15. What is meant in 2.2 'At the initial state, both the vanadium ions contents and the valence of the electrolytes are symmetry for the positive and negative sides. Hence, all the concentrations of vanadium ions on the two sides are the same at 50 SOC.'? What is the chemical state described here as 50 SOC? This thesis is confusing because '50 SOC' state is, normally, an ideal case of half-charged battery having xV^{2+}/xV^{3+} and 0V^{5+}/ 0V^{4+} (zero) at the anolyte side vs. xV^{5+}/xV^{4+} and 0V^{2+}/0V^{3+} (zero) at the catholyte side meaning different species concentrations on the two sides but equal concentration of each existing vanadium ion to each other; also this case excludes the preferential water transfer process.

Response: Thanks for your question. An ideal electrolytes state of VRFBs for the anolyte and catholyte should have a balanced content and valence of active species (specifically, the content of V^{2+} equal to that of VO_2^+; the contents of V^{3+} equal to that of VO^{2+}). However, the state of electrolytes on the positive and negative sides is broken with cycling due to the crossover, which results in capacity decay.

In Section 2.2 of the supporting information, at the initial stage means VRFBs before cycling. Hence, under this condition, the catholyte volume is 40 mL (1.70 M VO^{2+}), and the anolyte volume is 40 mL (1.70 M V^{3+}). Therefore, the contents and valences of active species in the catholyte and anolyte are balanced (40 mL 1.70 M V^{3+} $_{VS}$ 40 mL 1.70 M VO^{2+}; 0 M V^{2+} $_{VS}$ 0 M VO_2^+). In VRFBs, the crossover is an accumulated process and displays a slight effect on the capacity in one cycle (the coulombic efficiency of VRFB is larger than 98%, the corresponding capacity retention rate per cycle in 100 cycles is 99.53% based on Figs. 3(a-b)). Therefore, we can neglect the crossover effects when charging the VRFB from 0 to 50 SOC. It is to say, the content and concentration of the vanadium ions in the VRFBs are the same at 50 SOC (contents: $V^{3+} = VO_2^+ = V^{2+} = VO_2^+ = 0.5\times40\times1.7$ mM). In this work, we did not consider the water transfer effects on

capacity decay. Moreover, just as the response to Question 3, we have modified the description of Section 2.2 in the revised supporting information.

[In the revised supporting information, Section 2.1, Page S5, line 14]

The side reactions (Eqs. S1- S6) will occur when vanadium ions cross the membrane from one side to the other side, and the effect of crossover on vanadium ions' evolution in the charge/discharge process is depicted in Fig. S3. As shown in Fig. S3(a), although the crossover of V^{2+} and V^{3+} consumes different amounts of VO_2^+ in the catholyte, both result in the same amount of unavailable V^{2+} in the anolyte finally. Similarly, the crossover of VO^{2+} and VO_2^+ consumes different amounts of V^{2+} in the anolyte, both result in the same amount of unavailable VO_2^+ in the catholyte finally, as shown in Fig. S3(b). That means the accumulated amount of unavailable V^{2+} is a constant at a certain net flux of the electrolyte, no matter how much the vanadium ions (V^{2+}, V^{3+}, VO^{2+}, VO_2^+) contribute to the crossover separately. Moreover, the concentration and volume of the electrolyte increase on the positive side and decrease on the negative side with cycling[7] due to the much higher diffusion rate across the Nafion series membranes of V^{2+} than other vanadium ions.[3] The electrolytes' changing results in the surplus of V^{2+} on the negative side.[4,5] Therefore, we used the net flux of V^{2+} (the amount of vanadium ions (V^{2+}/ V^{3+}) crossover from the negative to the positive sides subtract the amount of vanadium ions (VO^{2+}/VO_2^+) crossover from the positive to the negative sides) to depict the effects of vanadium ions crossover on the accumulation process of unavailable V^{2+} in the following calculation for brevity.

$$V^{2+} + 2VO_2^+ + 2H^+ \rightarrow 3VO^{2+} + H_2O \quad (S1)$$

$$V^{2+} + VO^{2+} + 2H^+ \rightarrow 2V^{3+} + H_2O \quad (S2)$$

$$V^{3+} + VO_2^+ \rightarrow 2VO^{2+} \quad (S3)$$

Reactions for the vanadium ions transport from the positive to the negative sides:

$$VO_2^{+} + 2V^{2+} + 2H^{+} \rightarrow 3VO^{2+} + H_2O \tag{S4}$$

$$VO_2^{+} + V^{3+} \rightarrow 2VO^{2+} \tag{S5}$$

$$VO^{2+} + V^{2+} + 2H^{+} \rightarrow 2V^{3+} + H_2O \tag{S6}$$

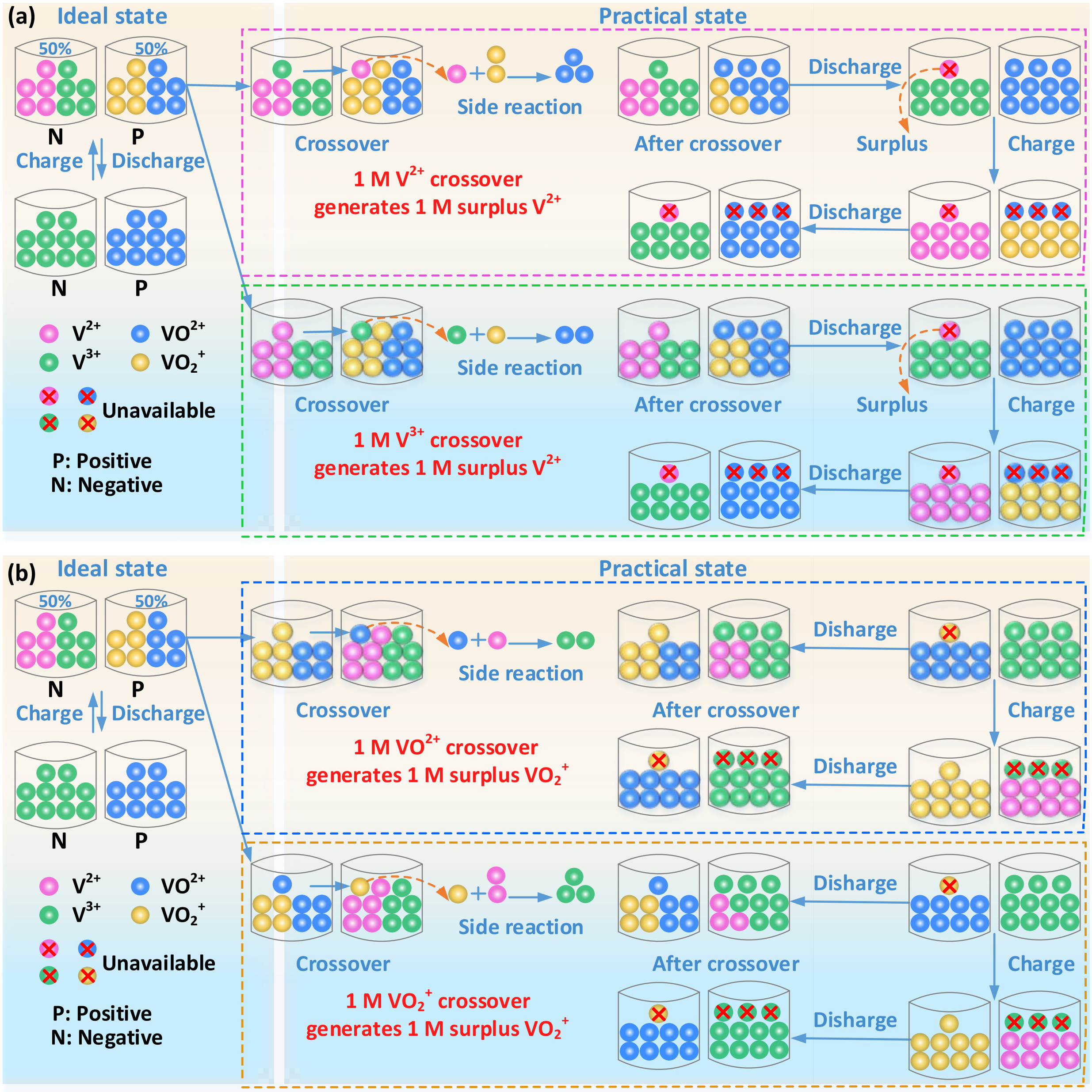

Fig. S3 Schematic of (a) V^{2+}/V^{3+} and (b) VO^{2+} /VO_2^{+} crossover effect on vanadium ions evolution in the charge/discharge process.

16. Some additional information shall be added to S2, S4 figures because it is hard to tell the difference between them from the description. What is the role of the viscosity measurements for the research and why are the trends are different?

Response: Thanks for your suggestion. Increasing the electrolytes' concentration leads to a high electrolyte viscosity. Therefore, the viscosity measurements are used to auxiliary demonstrate the concentration changes with cycling.

To be specific, Fig. S5 is used to demonstrate that the vanadium ions' concentration of the VRFB with $V^{3.5+}$ electrolyte varies with cycling which is also reflected in the change of electrolytes' viscosity. Fig. S7 is used to present the increasing hydraulic gradient between the two sides of the membrane with cycling. The hydraulic gradient drives the net flux of the active species transport from the catholyte to the anolyte and leads to the discharge capacity of Ox-C increasing with cycling after 300 cycles (Fig. 3(c)). We have added the related description under Fig. S2 and Fig. S4 in the revised supporting information. Details as below：

[In the revised supporting information, Section 3, Page S12, line 10]

Fig. S5 shows that the viscosity of electrolytes in the catholyte increases rapidly in the first 100 cycles and then slows down the increasing rate, which is consistent with the change in electrolytes' concentration with cycling. Similarly, the viscosity of electrolytes in the anolyte presents the same trend as that of the electrolytes' concentration change with cycling.

[In the revised supporting information document, Section 3, Page S13, line 14]

In Fig. S7, Ox-C represents the VRFB in which the anolyte is continually oxidized with air during cycling. Fig. S7 shows that the gap of viscosity between the anolyte and catholyte of Ox-C increases with cycling, which increases the amount of vanadium ions transport from the catholyte

to anolyte by convection and diminishes the lack of V^{3+} in the anolyte. Thus, the capacity of Ox-C increases with cycling after 300 cycles due to the increment of active species in the anolyte.

17. A table with all the settings and experimental data obtained shall be added.

Response: Thanks for your suggestion. We added an experimental settings table in the revised supporting information, detailed as shown below:

[In the revised supporting information, Section 1.6, Page S4, line 1]

Tab. S1 The setting parameters in VRFB tests.

Batteries	Catholyte	Anolyte	Operating voltage range	Current density (mA cm^{-2})
Ox-0	40 mL 1.7 M $V^{3.50+}$	40 mL 1.7 M $V^{3.50+}$	1.6-0.9 V	200
Ox-4	40 mL 1.7 M $V^{3.50+}$	40 mL 1.7 M $V^{3.50+}$	1.6-0.9 V	200
Ox-C	40 mL 1.7 M $V^{3.50+}$	40 mL 1.7 M $V^{3.50+}$	1.6-0.9 V	200
Ox-0-remixed	Ox-0 after 400 cycles	Ox-0 after 400 cycles	1.6-0.9 V	200
Ox-4-remixed	Ox-4 after 400 cycles	Ox-4 after 400 cycles	1.6-0.9 V	200
Ox-C-remixed	Ox-C after 400 cycles	Ox-C after 400 cycles	1.6-0.9 V	200
$V^{3.50+}$	40 mL 1.7 M $V^{3.50+}$	40 mL 1.7 M $V^{3.50+}$	1.6-0.9 V	200
$V^{3.68+}$	40 mL 1.7 M $V^{3.68+}$	40 mL 1.7 M $V^{3.68+}$	1.6-0.9 V	200
Fully discharge	----	----	0 V	312.5-200-125-62.5-50-18.75

18. Tab. S2: in order to make the available capacity calculations correct, you will need to change the metrics as (A h / L) as long as the system volume is not introduced and to add the formula explaining the values taken for the broad auditory of the journal that might not know the numbers used. Also, consider using 'mol L^{-1}' or 'M' through the article.

Response: Thanks for your suggestion. We have changed the capacity metrics to Ah L^{-1} in Fig. 2 and Tabs. S3-S4 and added the meaning of numbers used in the calculation. The metrics of M are also unified through the article in the revised manuscript and supporting information.

19. A part explaining the V^{2+} oxidizing process from the experimental setup perspective shall be added, especially in the case of Ox-C experiment. As for me, it raises a question of why did not all the V^{2+} oxidize? I would expect an accelerated capacity decay to a near-zero values in the case of a continuous air flow through the anolyte during the cycling.

Response: Thanks for your suggestion. This work used two different ways to oxidize V^{2+} with air. The one is oxidizing V^{2+} after a full discharge (Ox-4), and the other is oxidizing V^{2+} with air continually during the charge/discharge cycling (Ox-C). In the first method, a gradient decreasing current density (312.5-200-125-62.5-50-18.75 mA cm^{-2}) is applied to discharge the VRFBs to 0 SOC and avoid excessive oxidization of V^{2+}. Then, blow air into the anolyte tank, ensure the pipe is inserted into electrolytes (Fig. S2(a)) to accelerate the oxidization process and ensure V^{2+} is oxidized completely. Moreover, a sealing film (PARAFILM@, USA) is used to avoid the electrolyte splash. This process continues for 6 hours to ensure the oxidization of V^{2+} with air completely. For the continued oxidization of V^{2+} with air, we just loosen the sealing cap of the anolyte tank and allow air exchanges slowly inside and outside the tank after the oxygen in the tank is consumed by V^{2+}, as shown in Fig. S2(b). We have added Section 1.7, named Oxidizing electrolytes, in the revised supporting information. Detailed is shown below.

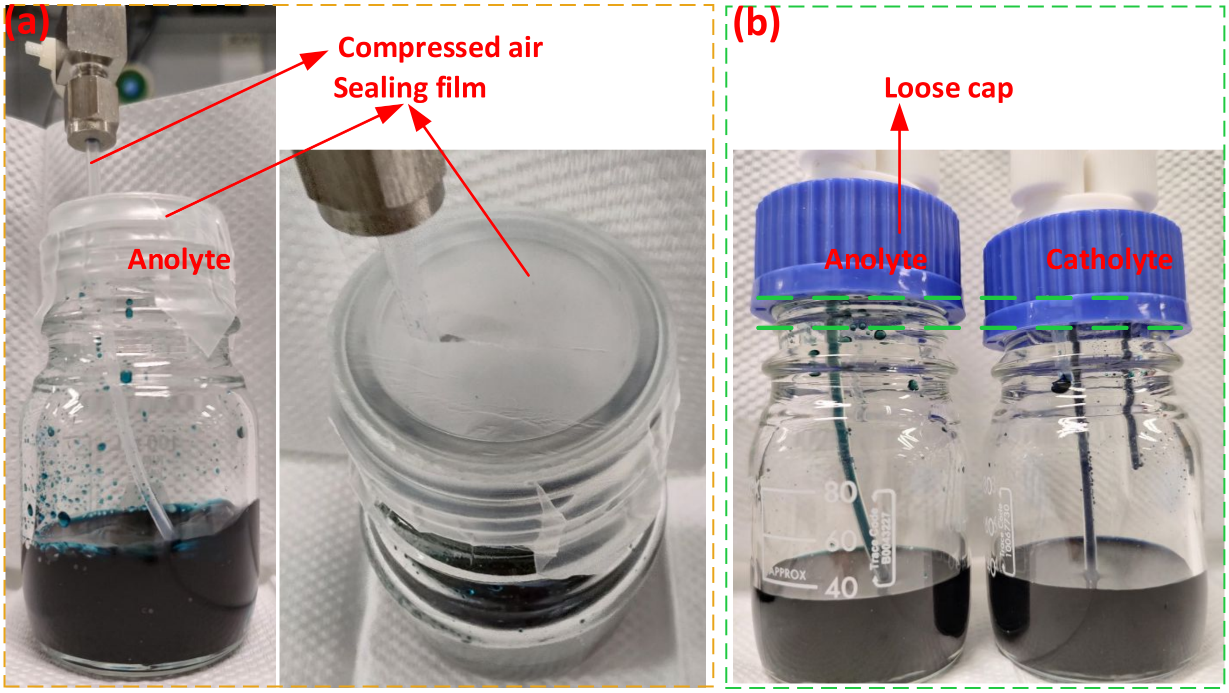

Fig. S2 Oxidization method of the unavailable V^{2+} in the anolyte. (a) Oxidizing V^{2+} with air at the end of a full discharge. (b) Oxidizing V^{2+} with air continually in the charge/discharge process.

In this work, oxidizing the unavailable V^{2+} with air is used to increase the active species of V^{3+} and make the content of V^{3+} closer to that of VO^{2+}. Hence, the VRFB cannot discharge if we oxidize all V^{2+} with air due to the lack of V^{2+} in the anolyte. Meanwhile, the VRFB cannot charge due to the lack of VO^{2+} in the catholyte, as shown in Fig. R2. Therefore, the discharge capacity is sharply down to 0 when we oxidize all V^{2+} with air (Fig. R2). Notably, a 50 mA cm^{-2} is applied on the VRFB before oxidizing the V^{2+} with air to ensure most of the V^{2+} is oxidized by air.

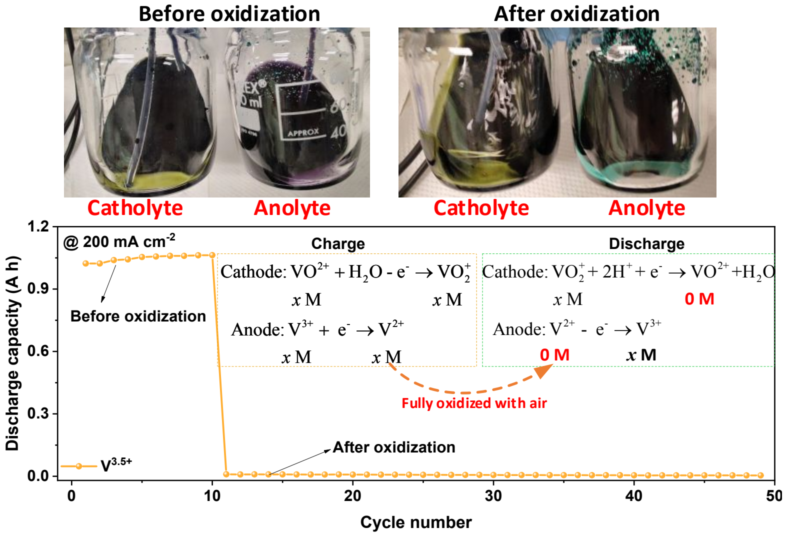

Fig. R2 The discharge capacity of VRFB before and after oxidizing V^{2+} with air completely.

As shown in Fig. R2, the capacity decay will be profoundly accelerated if excessive V^{2+} is oxidized with air during the charge/discharge process. Therefore, an experiment with compressed air blowing into the anolyte tank continually during the charge/discharge process is conducted, as shown in Fig. R3(a).

In Figs. R3(b-c), $V^{3.50+}$ means the VRFB with conventional electrolyte ($V^{3.50+}$) and carries out the charge/discharge with N_2 protection, and $V^{3.50+}$-air means the VRFB with conventional electrolyte ($V^{3.50+}$) and carries out the charge/discharge with N_2 protection in the first 30 cycles and then blow the compressed air into the anolyte tank during the charge/discharge cycling. Fig. R3(b) shows that $V^{3.50+}$ presents the same trend in the discharge capacity as that of $V^{3.50+}$-air in the first 30 cycles. However, the discharge capacity of $V^{3.50+}$-air increases in a few cycles after blowing compressed air into the anolyte tank because the accumulated unavailable V^{2+} is oxidized to V^{3+}, which is consistent with this work. Then, the discharge capacity rapidly decreases after 35 cycles due to the excessive oxidization of V^{2+} with air. The excessive oxidization of V^{2+} with air decreases the content of V^{2+} in the discharge process and results in lower coulombic efficiency and voltage

efficiency, as shown in Fig. R3(c). In Fig. R3(c), the rise of voltage efficiency at 63 cycles is caused by the minimal capacity (close to 0), which results in a significant fluctuation. The fluctuation is also reflected in the coulombic efficiency of $V^{3.5+}$-air, as shown in R3(c).

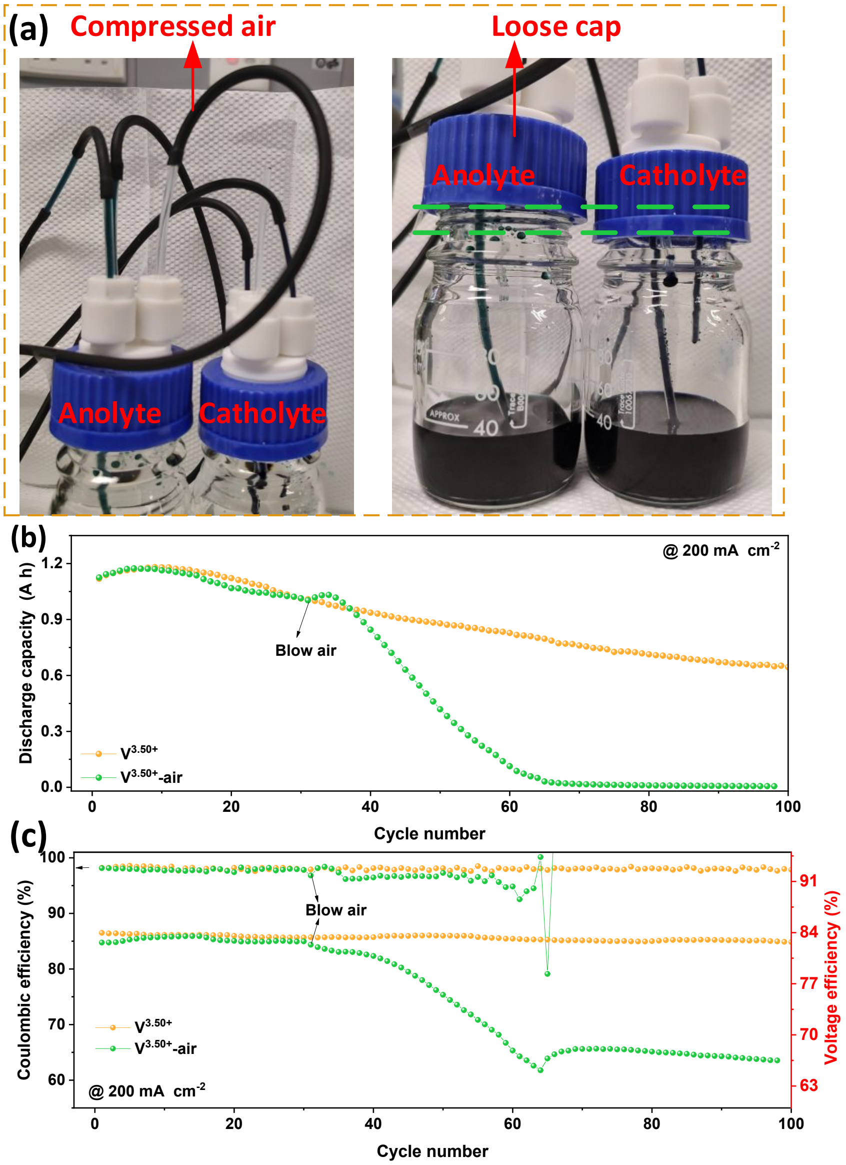

Fig. R3 VRFB performance with air flow continuously during the charge/discharge process. (a) The setup of compressed air blowing in the anolyte tank. The comparison of (b) discharge capacity and (c) coulombic/voltage efficiency before and after blowing air into the anolyte tank.

[In the revised supporting information, Section 1.7, Page S4, line 16]

1.7 Oxidizing V^{2+} with air

This work used two different ways to oxidize V^{2+} with air. One is oxidizing V^{2+} after a full discharge (Ox-4), and the other is oxidizing V^{2+} with air continually during the charge/discharge cycling (OX-C). In the first method, a gradient decreasing current density (312.5-200-125-62.5-50-18.75 mA cm^{-2}) is applied to discharge the VRFBs to 0 SOC and avoid excessive oxidization of V^{2+}. Then, blow air into the negative electrolyte tank and ensure the pipe is inserted into electrolytes (Fig. S2(a)) to accelerate the oxidization process and ensure V^{2+} is oxidized completely. Moreover, a sealing film (PARAFILM@, USA) is used to avoid the electrolyte splash. This process continues 6 hours to ensure the oxidization of V^{2+} with air completely. For the continued oxidization of V^{2+} with air, we just loosen the negative tank's sealing cap and allow air exchanges slowly inside and outside the tank after the oxygen in the tank is consumed by V^{2+}, as shown in Fig. S2(b).

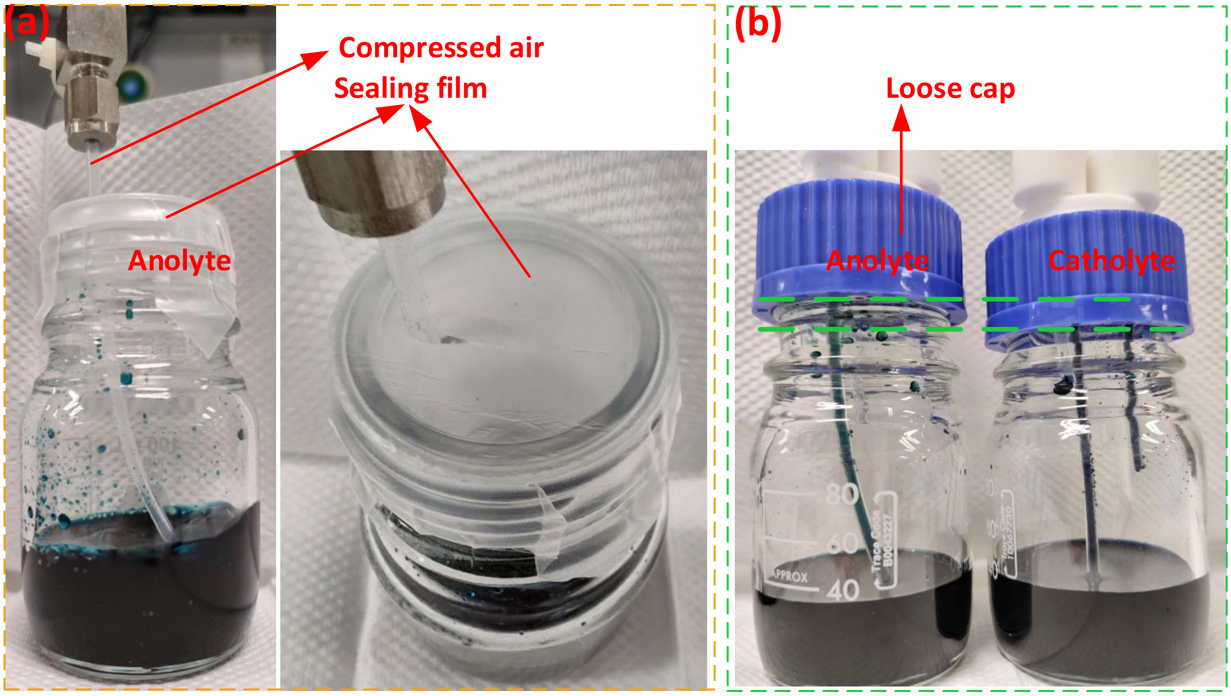

Fig. S2 Oxidization method of the unavailable V^{2+} in the anolyte. (a) Oxidizing V^{2+} with air at the end of a full discharge. (b) Oxidizing V^{2+} with air continually in the charge/discharge process.

20. The current density of 200 mA/cm2 is very high and reduces crossover significantly, while the research is exactly about this process. I would suggest such an experiment with 60 mA/cm current density, because it is hard to tell how close was the cycling to a symmetrical one from the SOC perspective, meaning asymmetric cycling, which is also a way of crossover control. In other words, the research might be considered as an addition to the asymmetric cycling procedure which makes the article very specific and would not reflect the general idea.

Response: Thanks for your question. As a most promising flow battery energy storage technology, VRFB is very close to commercialization. Till now, the operating current density of VRFBs is 150-300 mA cm^{-2}.[1] 200 mA cm^{-2} is a typical parameter widely used to estimate the battery performance in single cell[2,3] and kW scale stacks.[4,5,6] Therefore, we investigated the cycling performance of VRFBs with the proposed method under 200 mA cm^{-2} in this work.

The current density significantly affects the operating SOC of VRFBs in the charge/discharge process, which also influences the capacity decay in round charge/discharge cycling.[7] However, as the response to Question 3, although the crossover of V^{2+} and V^{3+} consumes a different amount of VO_2^+ on the positive side, both result in the same amount of unavailable V^{2+} on the negative side finally (Fig. S3(a). Similarly, the crossover of VO^{2+} and consuming different amounts of V^{2+} on the negative side result in the same amount of unavailable on the positive side, as shown in Fig. S3(b). That means the accumulated amount of unavailable V^{2+} is a constant at a certain net flux of electrolyte, no matter how much the vanadium ions (V^{2+}, V^{3+}, VO^{2+}, VO_2^+) contribute to the crossover separately. Moreover, the concentration and volume of the electrolyte increase on the positive side and decrease on the negative side with cycling[8] is a general result and was widely

reported.[9,10] Therefore, in this work, we used the net flux of V^{2+} (the amount of vanadium ions (V^{2+}/ V^{3+}) crossover from the negative to the positive sides subtract the amount of vanadium ions (VO^{2+}/VO_2^+) crossover from the positive to the negative sides) to depict the effects of vanadium ions crossover on the accumulation process of unavailable V^{2+} in the calculation for brevity.

Therefore, the method proposed in this work suits all the capacity decay caused by the crossover of the VRFBs equipped with Nafion series membranes. Reducing the operating current density of VRFBs will accelerate the crossover and the accumulation of unavailable V^{2+} in the anolyte under the same cycle numbers. Hence, the capacity improvement of the VRFB operating at 60 mA cm^{-2} should be larger than that running at 200 mA cm^{-2} using the proposed methods in this work.

(1) Zhang K, Yan C, Tang A. Interfacial co-polymerization derived nitrogen-doped carbon enables high-performance carbon felt for vanadium flow batteries[J]. *J Mater Chem A*, 2021, 9(32): 17300-17310. DOI: 10.1039/D1TA03683A

(2) Ding C, Zhang H, Li X, Liu T, and Xing F. Vanadium flow battery for energy storage: prospects and challenges[J]. *J Phys Chem Lett*, 2013, 4(8): 1281-1294. DOI:10.1021/jz4001032

(3) Vinco J H, da Cunha Domingos A E E, Espinosa D C R, Tenerio J A S, and Baltazar P G . Unfolding the Vanadium Redox Flow Batteries: An indeep perspective on its components and current operation challenges[J]. *J Energy Storage*, 2021, 43: 103180. DOI:10.1016/j.est.2021.103180

(4) Zou T, Shi X, Yu L. Study on energy loss of 35 kW all vanadium redox flow battery energy storage system under closed-loop flow strategy[J]. *J Power Sources*, 2021, 490: 229514. DOI: 10.1016/j.jpowsour.2021.229514

(5) Dai Q, Xing F, Liu X, Shi D, Deng C, Zhao Z, and Li X. High-performance PBI membranes for flow batteries: from the transport mechanism to the pilot plant[J]. *Energ Environ Sci*, 2022, 15(4): 1594-1600. DOI: 10.1039/D2EE00267A

(6) Li T, Xing F, Liu T, Sun J, Shi D, Zhang H, and Li X. Cost, performance prediction and optimization of a vanadium flow battery by machine-learning[J]. *Energ Environ Sci*, 2020, 13(11): 4353-4361. DOI: 10.1039/D0EE02543G

(7) Luo Q, Li L, Wang W, Nie Z, Wei X, Li B, Chen B, Yang Z, and Sprenkle V. Capacity decay and remediation of nafion-based all-vanadium redox flow batteries. *ChemSusChem*. 2013;6(2):268–74. DOI: 10.1002/cssc.201200730

(8) Sun C, Chen J, Zhang H, Han X, and Luo Q. Investigations on transfer of water and vanadium ions across Nafion membrane in an operating vanadium redox flow battery[J]. *J Power Sources*, 2010, 195(3): 890-897. Doi:10.1016/j.jpowsour.2009.08.041

(9) Song Y, Li X, Xiong J, et al. Electrolyte transfer mechanism and optimization strategy for vanadium flow batteries adopting a Nafion membrane[J]. *J Power Sources*, 2020, 449: 227503. DOI: 10.1016/j.jpowsour.2019.227503

(10) Tang A, Bao J, Skyllas-Kazacos M. Dynamic modelling of the effects of ion diffusion and side reactions on the capacity loss for vanadium redox flow battery. *J Power Sources*. 2011;196(24):10737–47. DOI: 10.1016/j.jpowsour.2016.02.018

21. Make a general revision. I could have missed something as there are a lot of issues with the clearance.

Response: Thanks for your suggestion. We have checked the supporting information carefully and corrected some issues in the revised document.

We have tried our best to answer the question and improve the manuscript. We also appreciate Editors/Reviewers' warm work and earnestly hope the correction will meet with approval. Once again, thank you very much for your comments and suggestions.

With best regards,

Sincerely,

Tianshou Zhao, Chair Professor

Department of Mechanical and Aerospace Engineering,

The Hong Kong University of Science and Technology

Clear Water Bay, Kowloon, Hong Kong, China

E-mail: metzhao@ust.hk

oc-2022-01112j.R2

Name: Peer Review Information for "An Electrolyte with Elevated Average Valence for Suppressing the Capacity Decay of Vanadium Redox Flow Batteries"

Second Round of Reviewer Comments

Reviewer: 2

Comments to the Author

The article is written very well, clear and straightfoward. Experimental data is described precisely and the overall quality of the paper is high. It proposes an original and counter-intuitive approach in solving the capacity fade problem: it demonstrates a positive impact of an increased averaged electrolyte valence on the capacity losses during the cycling. I think, the article can have a significant impact on the VRFB technology because it is standard now for both laboratories and manufacturers to work with 3.5 averaged valence electrolyte. Moreover, there are researches trying to reduce the rebalanced electrolyte's averaged valence back to 3.5 value or to decrease its growth.

Speaking of the temperature caused oscillations (Fig.4), those are not interfering with the method demonstration, moreover, they are closer to the real-life conditions than if it was in the thermostat.

I would only suggest changing the direction of the arrows Fig.S3(b) at the discharge stage because they seem to be mistakenly reversed. There is also a question if it is correct to use "M" symbol there because the figure assumes "mol" transfer. Not mole/liter (concentration) but the amount of substance.

There is also a typo on page 2: "Nafion series membranes are the most widely used proton exchange membrane in VRFBs.". I guess, it was meant membraneS.

Author's Response to Peer Review Comments:

Thank you for your and reviewers’ comments and affirmation of our work. The point-to-point responses are uploaded as an attachment.

Dear Editors and Reviewers,

Thank you for your and reviewers' comments and affirmation to our manuscript entitled "An Electrolyte with Elevated Average Valence for Suppressing the Capacity Decay of Vanadium Redox Flow Batteries" (oc-2022-01112j.R1), which are very valuable and helpful for improving this paper. We considered these comments seriously and revised the manuscript and supporting information document based on them. The point-to-point responses to the comments are listed as follows:

Reviewer(s)' Comments to Author:

Reviewer: 2

Recommendation: Publish in ACS Central Science after minor revisions noted.

Comments:

The article is written very well, clear and straightforward. Experimental data is described precisely and the overall quality of the paper is high. It proposes an original and counter-intuitive approach in solving the capacity fade problem: it demonstrates a positive impact of an increased averaged electrolyte valence on the capacity losses during the cycling. I think, the article can have a significant impact on the VRFB technology because it is standard now for both laboratories and manufacturers to work with 3.5 averaged valence electrolyte. Moreover, there are researches trying to reduce the rebalanced electrolyte's averaged valence back to 3.5 value or to decrease its growth.

Speaking of the temperature caused oscillations (Fig.4), those are not interfering with the method demonstration, moreover, they are closer to the real-life conditions than if it was in the thermostat.

1. *I would only suggest changing the direction of the arrows Fig.S3(b) at the discharge stage because they seem to be mistakenly reversed. There is also a question if it is correct to use "M" symbol there because the figure assumes "mol" transfer. Not mole/liter (concentration) but the amount of substance.*

Response: Thanks for your valuable suggestions. We are very sorry for the typos in Fig. S3(b). We have corrected the direction of arrows and mended the unit "M" to "mol" in the revised supporting information document.

2. *There is also a typo on page 2: "Nafion series membranes are the most widely used proton exchange membrane in VRFBs.". I guess, it was meant membraneS.*

Response: Thanks for your valuable suggestion. We are very sorry for the typo. We have corrected it in the revised manuscript.

Besides, the formatting of the manuscript is also revised as the journal's requirements.

We very appreciate Editors/Reviewers' warm work and earnestly hope the correction will meet with approval. Once again, thank you very much for your comments and suggestions.

With best regards,

Sincerely,

Tianshou Zhao, Chair Professor

Department of Mechanical and Aerospace Engineering,

The Hong Kong University of Science and Technology

Clear Water Bay, Kowloon, Hong Kong, China

E-mail: metzhao@ust.hk
